# Barriers and facilitators to yoga practice among people living with arthritis: a qualitative systematic review

**DOI:** 10.1007/s00296-025-06037-5

**Published:** 2025-12-17

**Authors:** Isha Biswas, Patricia Egwumba, Catrin Evans, Sarah Lewis, Kaushik Chattopadhyay

**Affiliations:** 1 Lifespan and Population Health, School of Medicine, University of Nottingham, Nottingham, UK; 2 School of Health Sciences, University of Nottingham, Nottingham, UK; 3 The Nottingham Centre for Evidence-Based Healthcare: A JBI Centre of Excellence, Nottingham, UK

**Keywords:** Arthritis, Qualitative research, Systematic review, Yoga

## Abstract

The global burden of arthritis is high and increasing. Systematic reviews suggest that yoga, an ancient mind-body discipline, may help in arthritis treatment. This systematic review aimed to synthesise the barriers and facilitators to yoga practice in people with arthritis. JBI methodological guidance for qualitative systematic reviews was followed. MEDLINE, Embase, CINAHL Plus, PsycInfo, AMED, and Web of Science were searched to identify published studies, and ProQuest Dissertations and Theses for unpublished studies. Databases were searched until 07 November 2024, with no language restrictions. Study screening, assessment of methodological quality, and data extraction were completed independently by two reviewers. Data were synthesised using a meta-aggregative approach. Of 1330 identified records, nine articles, representing eight studies, were included in the review. All studies were conducted in high-income countries (the USA, UK, and New Zealand), with a majority of female participants. Methodological quality ranged from moderate to high; six of the eight studies met at least seven of the ten quality assessment criteria. 112 findings were extracted from the articles and grouped into 20 categories based on similarity in meaning. These were formulated into five synthesised findings: (i) Yoga, arthritis, and the body: the anticipated and experienced impacts of yoga on physical well-being influenced yoga practice; (ii) Yoga, arthritis, and the mind: levels of motivation and perceived impact on mental well-being influenced yoga practice; (iii) Yoga, arthritis, and the mind-body impact: yoga’s mind-body benefits supported coping with arthritis and encouraged continued practice; (iv) Yoga, arthritis, and session accessibility and structure: factors related to session accessibility and structure influenced engagement with yoga; and (v) Yoga, arthritis, and the session environment: a supportive social environment in yoga sessions impacted yoga practice. Each synthesised finding revealed a range of barriers and facilitators to yoga practice in people with arthritis. Within the included studies, there appeared to be more facilitators than barriers, suggesting that yoga could be a valuable addition to arthritis treatment. Future interventions to support yoga practice in this group should promote these facilitators and address the barriers to ensure successful implementation. *PROSPERO registration number*: CRD42023483350.

**Supplementary Information:**

The online version contains supplementary material available at 10.1007/s00296-025-06037-5.

## Introduction

Arthritis is a set of chronic musculoskeletal conditions affecting the joints [1]. It is generally characterised by joint pain, stiffness, inflammation, and deformity, and can lead to impaired joint function [1, 2]. Arthritis has significant physical and mental health impacts and limits participation in social activities [3–6]. It also leads to a considerable economic burden, such as costs for medical treatments, and indirect costs due to absenteeism from work and loss of work productivity [7, 8]. Eventually, the individual’s health-related quality of life is affected [8, 9]. Osteoarthritis and rheumatoid arthritis are two major contributors to the global burden of musculoskeletal conditions, affecting nearly 528 million and 18 million people, respectively [1, 10].

Western medical treatment typically includes the use of non-steroidal anti-inflammatory drugs (NSAIDs) for osteoarthritis and disease-modifying anti-rheumatic drugs (DMARDs) for rheumatoid arthritis [11, 12]. However, long-term medication use may have side effects (e.g., gastrointestinal toxicity) [12, 13]. In addition, non-pharmacological approaches to treatment (e.g., physical exercises) are also recommended for symptom relief [2, 11, 12]. Though beneficial, these approaches can be challenging due to high costs, inability to meet individualised needs, difficulty level, and injury concerns [14–17].

Yoga, an ancient mind-body practice originating in the Indian subcontinent, imparts a sense of well-being of the body and mind, and offers an alternative that may address these concerns [18, 19]. Its global popularity continues to rise, with nearly 300 million people practising it [20–22]. Yoga typically involves a gentle approach, requires minimal equipment, and can be practised with a low to moderate level of guidance, in indoor and outdoor settings [19, 23]. Systematic reviews and meta-analyses have shown that yoga is safe and can be beneficial in osteoarthritis and rheumatoid arthritis treatment [24–28]. Further, the American College of Rheumatology (ACR) and the European League Against Rheumatism (EULAR) guidelines conditionally recommend yoga as a complementary approach for the treatment of knee osteoarthritis and rheumatoid arthritis [29, 30].

Qualitative research can provide insights into why people with arthritis may, or may not, practice yoga. Such studies have explored the factors that impede (barriers) and encourage (facilitators) yoga practice among people with arthritis [31–33]. However, no systematic review on this topic has been conducted to date. Therefore, this systematic review aimed to synthesise the barriers and facilitators to yoga practice in people with arthritis. It is hoped that the review findings could be used to address the barriers through appropriate actions and promote the facilitators of yoga practice in people with arthritis.

## Methods

The review adhered to JBI methodological guidance on systematic reviews of qualitative evidence [34]. It was reported according to the ‘Enhancing Transparency in Reporting the Synthesis of Qualitative Research’ (ENTREQ) statement and the Preferred Reporting Items for Systematic Reviews and Meta-analyses (PRISMA) guideline [35, 36]. This review protocol was registered with PROSPERO (CRD42023483350).

### Inclusion criteria

*Participant*: This review included studies conducted among adults (aged ≥ 18 years) diagnosed with arthritis of any type. No restrictions on diagnostic criteria were applied.

*Phenomena of interest*: This review included studies that explored the knowledge, experiences, attitudes, understandings, perceptions, or perspectives that may act as barriers and facilitators to yoga practice.

*Context*: This review considered studies undertaken in any global context and setting (e.g., community, primary care, secondary care, or tertiary care).

*Study design*: This review considered studies that had qualitative data, including, but not limited to, designs such as phenomenology, ethnography, action research, case studies, grounded theory, and feminist research. Other study designs, such as mixed methods, quasi-experimental, and cross-sectional descriptive studies, reporting relevant qualitative data, were also included.

### Databases and search strategy

Six electronic databases were searched from their inception dates until 07 November 2024 to locate published studies: (i) MEDLINE (Ovid; from 1946), (ii) Embase (Ovid; from 1974), (iii) CINAHL Plus (EBSCOhost; from 1937), (iv) PsycInfo (Ovid; from 1806), (v) AMED (Ovid; from 1985), and (vi) Web of Science (from 1900). In addition, ProQuest Dissertations and Theses were searched for unpublished studies. The search strategies were developed for all the databases in consultation with an experienced research librarian at the University of Nottingham (UK). The search strategy was initially developed for MEDLINE and then adapted as necessary across other databases. “Yoga” and “arthritis” search concepts were based on the search strategies used in previous relevant systematic reviews [25, 26]. Predesigned database-specific search filters were used for the “qualitative study design” concept, where possible [37]. No language restrictions were applied. The search strategies are detailed in Appendix 1. The reference lists of all the included studies were screened for additional studies.

### Study screening and selection

Following the searches, all identified citations were collated and uploaded into EndNote X9 [38] and de-duplicated. The remaining records were then imported into Rayyan [39] to facilitate the title and abstract screening process, undertaken by two independent reviewers (IB and PE). Studies identified as potentially eligible or those without an abstract were retrieved in full text, and their details were imported into the JBI System for the Unified Management, Assessment and Review of Information (JBI SUMARI) [40]. The full text of the studies was then assessed in detail against the inclusion criteria by the two independent reviewers. Any disagreements between the two reviewers at each stage of the study selection process were resolved through discussion or by involving a senior reviewer (SL/KC) if a consensus was not reached.

### Assessment of methodological quality

The two independent reviewers assessed all eligible studies using the standardised critical appraisal checklist for qualitative research incorporated within JBI SUMARI [40]. The checklist uses a series of criteria that can be scored as being met (yes), not met (no), unclear, or, where appropriate, not applicable (n/a) to the particular study. They went through each criterion and commented on it. Any disagreements between reviewers were resolved through discussion or by involving a senior reviewer. All studies, regardless of their methodological quality, underwent data extraction and synthesis where possible.

### Data extraction

The two independent reviewers extracted data using the standardised data extraction tool incorporated within JBI SUMARI [40]. Any disagreements were resolved through discussion or involving the third reviewer. The following details were extracted: author and year of publication, country, phenomena of interest, yoga delivery setting, participant recruitment setting, qualitative research methodology, study design, sample size and participant characteristics (type of arthritis, age, sex, and disease duration), data collection methods, and data analysis technique. Next, findings (i.e., extracted themes from included studies), with relevant illustrations (i.e., quotes or supportive data cited in the included studies for each finding), were extracted. The findings and illustrations were the actual verbatim words of the study authors and participants, respectively. Each finding was then assigned a level of credibility: unequivocal (U) (i.e., evidence beyond a reasonable doubt), credible (C) (i.e., evidence that was open to challenge), or not supported (NS) (i.e., findings were not supported by the data) [40].

### Data synthesis

All authors were involved in data synthesis. The extracted study details were first narratively synthesised. Then, study findings were pooled using JBI SUMARI, following a meta-aggregation approach [41]. Initially, the lead reviewer (IB) grouped findings assigned as either unequivocal or credible into categories based on similarity in meaning and concept through discussions with another reviewer (PE). Each finding was labelled, and the related or similar ones were grouped under a representative name. This iterative process continued until all authors (IB, PE, CE, SL, and KC) reached consensus to ensure that the findings were placed under appropriate categories. To interpret the categories as barriers or facilitators, patterns in the data and their relevance to the review objective were analysed. This process helped identify factors influencing yoga practice in people with arthritis, providing a clearer understanding of both barriers and facilitators. The lead reviewer refined the categories and aggregated them into synthesised findings through repeated discussions with other senior reviewers (CE, SL, and KC), formulating statements to represent each.

## Results

### Study inclusion

**Fig. 1:**
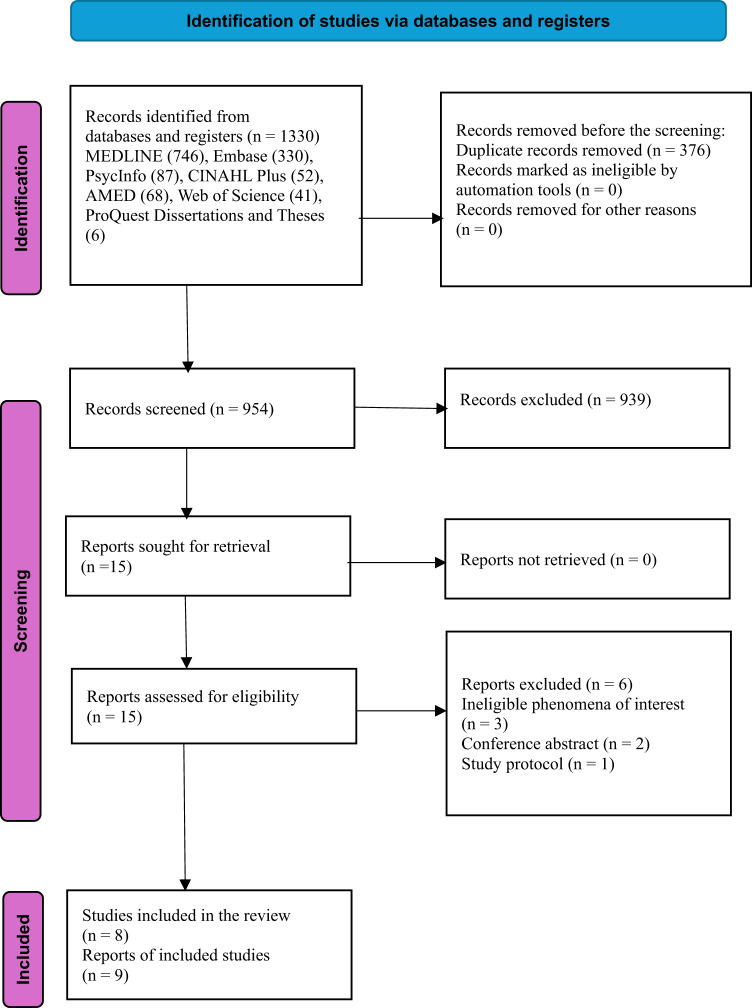
PRISMA flow diagram for included studies from searches of databases and registers only

The study selection process is detailed in the PRISMA flowchart as shown in Fig. 1. 1330 records were identified through the literature search. After removing duplicate records and title and abstract screening, 15 articles were retrieved for full-text screening. A total of six articles were excluded after full-text screening. The most common reasons were ineligible phenomena of interest (*n* = 3), conference abstract (*n* = 2), and study protocol (*n* = 1). Nine articles, representing eight studies, were included in the review [31–33, 42–47]. Appendix 2 provides information on excluded studies and the reasons for exclusion. No additional articles were identified from citation searching.

### Characteristics of included studies

Table 1 describes the characteristics of the eight included studies [31–33, 42–47]. Two articles originated from the same study [33, 42]. Each had different aims and data analysis techniques but were based on the same set of qualitative interview data; hence, they were considered a single study in this review [33, 42]. The included studies were published over 12 years from 2010 to 2022 [31–33, 42–47]. All studies were conducted in high-income countries, specifically, New Zealand [47], the UK [44], and the USA [31–33, 42, 43, 45, 46]. All studies explored views, experiences, perceptions, or perspectives of people with arthritis regarding yoga practice. Participants were recruited solely from community settings in three studies [31, 43, 46], presumed secondary care settings in three studies [44, 45, 47], and tertiary care settings in one study [32]. Additionally, in one study, participants were recruited from both presumed secondary care and community settings [33, 42]. In six studies, yoga interventions were delivered as group sessions in community settings [31–33, 42, 43, 45, 46], including two studies where participants also practised individually at home [31, 43]. In one study, the yoga intervention was delivered in one-on-one sessions, presumably in a secondary care setting and also practised individually at home [44]. Another study explored insights regarding a potential yoga intervention but did not include its actual delivery [47]. Regarding qualitative research methodology, seven studies employed descriptive methodologies [31, 32, 43–47]. One study used both ethnographic [42] and phenomenological [33] methodologies (reported in two separate articles). Four studies solely used a qualitative study design [31, 32, 45, 47], two used mixed methods study designs [33, 42, 44], one used quasi-experimental [46], and one used a cross-sectional descriptive study design [43], allowing extraction of relevant qualitative data. The number of study participants ranged from 5 [33, 42] to 31 participants [43]. Where reported, the mean age of the participants ranged from 28 years [33, 42] to 72 years [43]. Five studies included both male and female participants [33, 42, 44–47], and three included only females [31, 32, 43]. The mean disease duration ranged from eight years [44] to 21 years [45]. Data collection methods primarily included interviews [31–33, 42–45], one of which used telephone interviews [45]. The remaining studies used focus group discussions for collecting data [46, 47]. Data analysis techniques included thematic analysis [44–47], content analysis [31, 32, 43], and phenomenological [33] and ethnographic content analysis [42] (the latter two were applied in one study, reported as two distinct articles).

**Table 1:** Characteristics of included studies

Author and year of publication	Country	Phenomena of interest	Participant recruitment setting	Yoga delivery setting	Qualitative research methodology	Study design	Sample size and participant characteristics (type of arthritis, age in years [mean (SD or range)], sex, disease duration in years [mean (SD or range)])	Data collection methods	Data analysis technique
Evans, 2010 [42]	USA	Exploring the range and depth of experiences regarding an IY programme	Secondary care? and community	Group sessions in community	Ethnography	Mixed methods	5 participants with RA Age: 28 (3) 4 females and 1 male Disease duration: 16 (8.5)	Face-to-face semi-structured interviews	Ethnographic content analysis
Evans, 2011 [33]	USA	Exploring potential mechanisms of change as a result of participating in an IY programme	Secondary care? and community	Group sessions in community	Phenomenology	Mixed methods	5 participants with RA Age: 28 (24–31) 4 females and 1 male Disease duration: 16 (8–28)	Face-to-face semi-structured interviews	Phenomenological analysis
Park, 2011 [46]	USA	Exploring the pain reduction, well-being, mood, and functional benefits after participation in a chair yoga programme	Community	Group sessions in community	Qualitative descriptive approach	Quasi-experimental (included qualitative approach)	7 participants with OA Age: NR Females and males: NR Disease duration: NR	Face-to-face focus group discussions	Thematic analysis
Ward, 2011 [47]	New Zealand	Exploring views regarding the suitability of yoga as a non-pharmacological option in the management of RA	Secondary care?	N/A	Qualitative descriptive approach	Qualitative	22 participants with RA Age range: 26–73 19 females and 3 males Disease duration: 16	Face-to-face focus group discussions	Thematic analysis
Cheung, 2015 [43]	USA	Identifying the barriers and motivations to yoga practice and exploring the experiences of home-based yoga practice	Community	Group sessions in community and individual practice at home	Qualitative descriptive approach	Cross-sectional descriptive (included qualitative approach)	31 participants with knee OA Age: 72 (6) All females Disease duration: NR	Face-to-face interviews and videotapes of home practice	Content analysis
Greysen, 2017 [45]	USA	Exploring community yoga practice characteristics and perceptions of how and why yoga is practised and factors influencing yoga participation as a symptom management strategy in RA	Secondary care?	Group sessions in community	Qualitative descriptive approach	Qualitative	17 participants with RA Age: 56 (11) 16 females and 1 male Disease duration: 21 (11)	Telephonic structured interviews	Thematic analysis
Middleton, 2017 [32]	USA	Identifying the barriers and facilitators to an adapted HY programme among adults from minority groups (English or Spanish speaking)	Tertiary care	Group sessions in community	Qualitative descriptive approach	Qualitative	12 participants with either OA or RA Age: 64 (33–65) All females Disease duration: NR	Face-to-face semi-structured interviews	Content analysis
Cartwright, 2020 [44]	UK	Exploring experiences and perspectives of an adapted YT intervention based on principles of Viniyoga	Secondary care?	One-on-one sessions in secondary care? and individual practice at home	Qualitative descriptive approach	Mixed methods	10 participants with RA Age: 54 (13) 9 females and 1 male Disease duration: 8 (6)	Face-to-face semi-structured interviews	Thematic analysis
Cheung, 2022 [31]	USA	Uncovering the experience and perspectives of long-term practice of a structured HY programme and identifying the barriers to and facilitators of adherence to the programme	Community	Group sessions in community and individual practice at home	Qualitative descriptive approach	Qualitative	28 participants with knee OA Age: 71 (8) All females Disease duration: NR	Face-to-face semi-structured interviews and focus group discussions	Inductive content analysis

? Unclear, *HY* Hatha yoga, *IY* Iyengar yoga, *N/A* Not applicable, *NR* Not reported, *OA* Osteoarthritis, *RA* Rheumatoid arthritis, *SD* Standard deviation, *UK* United Kingdom, *USA* United States of America, *YT* Yoga therapy

### Methodological quality of included studies

The critical appraisal results of the eight included studies are presented in Table 2. Overall, the methodological quality ranged from moderate to high. None of the studies met al.l of the appraisal criteria. Six studies had “yes” responses to 7 or more questions on the checklist [31, 32, 44, 45, 47, 48]. All studies met the criteria for Q2, Q3, Q4, Q5, Q8, and Q10. However, three questions consistently yielded lower ratings (most often rated as “unclear”): inadequate reporting of congruity between the stated philosophical perspective and the research methodology (Q1); inadequate reporting of cultural or theoretical positioning of the researcher (Q6; and limited reporting of the influence of the researcher on the study and vice-versa (Q7). Ratings of “unclear” reflect gaps in reporting in the original papers, which prevented a definitive judgment.

**Table 2:** Critical appraisal of included studies

Study	Q1	Q2	Q3	Q4	Q5	Q6	Q7	Q8	Q9	Q10
Evans, 2010, 2011 [33, 42]	U	Y	Y	Y	Y	U	U	Y	Y	Y
Park, 2011 [46]	Y	Y	Y	Y	Y	U	U	Y	Y	Y
Ward, 2011 [47]	U	Y	Y	Y	Y	N	U	Y	U	Y
Cheung, 2015 [43]	U	Y	Y	Y	Y	U	U	Y	Y	Y
Greysen, 2017 [45]	U	Y	Y	Y	Y	U	U	Y	Y	Y
Middleton, 2017 [32]	U	Y	Y	Y	Y	Y	Y	Y	Y	Y
Cartwright, 2020 [44]	U	Y	Y	Y	Y	N	Y	Y	Y	Y
Cheung, 2022 [31]	U	Y	Y	Y	Y	U	U	Y	Y	Y
Total % of “Y”	13	100	100	100	100	13	25	100	88	100

Y = yes; N = no; U = unclear. For example, Q1 was rated “U” for Evans (2010, 2011) due to insufficient detail on philosophical perspective, making it difficult to determine if the research methodology aligned with the stated philosophical perspective. Q7 was rated “U” for Park (2011) because the study did not clearly discuss how researchers’ interactions with participants may have influenced the research process. Note: similar issues apply to other studies with U ratings

JBI critical appraisal checklist for qualitative research: Q1 Is there congruity between the stated philosophical perspective and the research methodology? Q2 Is there congruity between the research methodology and the research question or objectives? Q3 Is there congruity between the research methodology and the methods used to collect data? Q4 Is there congruity between the research methodology and the representation and analysis of data? Q5 Is there congruity between the research methodology and the interpretation of results? Q6 Is there a statement locating the researcher culturally or theoretically? Q7 Is the influence of the researcher on the research, and vice-versa, addressed? Q8 Are participants, and their voices, adequately represented? Q9 Is the research ethical according to current criteria or, for recent studies, and is there evidence of ethical approval by an appropriate body? Q10 Do the conclusions drawn in the research report flow from the analysis, or interpretation, of the data?

### Review findings

Table 3 depicts the meta-aggregation of findings. A total of 112 findings were extracted from nine articles, of which 111 were assessed as unequivocal and one as credible. There were no unsupported findings. These were grouped into 20 categories and further interpreted into five synthesised findings. Each synthesised finding, along with its categories, findings, and illustrations, is detailed in Appendices 3, 4, 5, 6, and 7. Table 4 shows the categories and their representative illustration(s).

**Table 3:** Meta-aggregation of findings

Synthesised finding	Category	Findings
Yoga, arthritis, and the body: The anticipated and experienced impacts of yoga on physical well-being influenced yoga practice in people with arthritis.	*Barriers*	30
	1a. Yoga practice seemed “an unknown territory” that might aggravate arthritis symptoms.	
	1b. Uncertainty about yoga’s benefits on arthritis symptoms discouraged yoga practice.	
	*Facilitators*	
	1c. Experiencing relief from arthritis symptoms as a result of yoga practice.	
	1d. Yoga seemed to elevate body awareness and physical vitality, translating into people’s daily lives.	
	1e. Yoga practice was perceived to reduce reliance on arthritis medication for pain relief.	
Yoga, arthritis, and the mind: Levels of motivation and perceived impact on mental well-being influenced yoga practice in people with arthritis.	*Barriers*	29
	2a. Wavering intrinsic motivations (concentration, self-efficacy, and self-assurance) negatively influenced yoga practice.	
	*Facilitators*	
	2b. High levels of intrinsic motivations (personal interest in yoga, prioritising health, self-efficacy, self-assurance, and self-confidence) facilitated yoga practice.	
	2c. Extrinsic motivations (doctor’s advice, reduced price of yoga sessions, improvement in other health conditions, exposure to yoga, and peer/family support) positively influenced yoga practice in arthritis.	
	2d. Perception of improvements in mental well-being could encourage yoga practice.	
Yoga, arthritis, and the mind-body impact: The experience of mind-body benefits of yoga fostered a positive outlook on coping with arthritis and encouraged ongoing engagement with yoga practice in people with arthritis.	*Facilitators*	23
	3a. Practising yoga gave people with arthritis a sense of empowerment, i.e., the ability to take control of their condition.	
	3b. Yoga was appreciated as a beneficial coping strategy for arthritis.	
	3c. Practising yoga changed the way people with arthritis viewed their condition, allowing them to rediscover themselves and regain a sense of normalcy in their lives.	
	3d. Consistent yoga practice was perceived as essential for achieving long-term holistic benefits for arthritis.	
Yoga, arthritis, and session accessibility and structure: Engagement with yoga practice was influenced by individually determined factors affecting access to sessions, as well as by the structural characteristics of the sessions.	*Barriers*	22
	4a. Juggling with logistical challenges to attending yoga sessions discouraged yoga practice.	
	4b. Unsatisfactory yoga experiences, including the difficulty level of the practices and discomfort, hindered yoga practice.	
	*Facilitators*	
	4c. Yoga’s adaptability and well-paced sessions to suit people’s needs and preferences in arthritis were perceived to be helpful.	
	4d. Availability of a yoga provider with positive qualities, knowledge, and professional training facilitated yoga practice.	
	4e. The provision of props and resources was perceived to offer confidence and security for yoga practice.	
Yoga, arthritis, and the session environment: A supportive social environment in yoga sessions, characterised by a welcoming space and meaningful connections, encouraged yoga practice in people with arthritis.	*Facilitators*	8
	5a. Safe and supportive space (in group sessions) and a “therapeutic” space (in one-to-one sessions) were considered important for yoga practice.	
	5b. Social connectedness in a group setting was perceived as a strong motivation for yoga practice.	
Total synthesised findings = 5	Total categories = 20	Total findings = 112

**Table 4:** Categories and their representative illustration(s)

Category	Representative illustration(s), study author, and page number
1a	“I saw people at gym doing it (yoga) and you’re like ‘yeah right’ I’m never going to be able to do that I’m not even gonna try…at first, I didn’t even consider it. You hear yoga and arthritis and you just don’t think the two mix.” (Evans, 2011, p4)
	‘‘I had to stop doing yoga because of my RA. I actually tried yoga again recently, but it caused a flare, so, I’ve got to stop doing that.” (Greysen, 2017, p490)
1b	“And in terms of the pain, I think some days I thought ‘‘Oh wow, it’s really working’’ and some days I don’t know. It’s hard to really tell.” (Evans, 2010, p910)
1c	“I am very happy because I have learned yoga…it is helping me feel better, sleep better, to stretch and that helps me in my arthritis and I forget that I have it/suffer from it.” (Middleton, 2017, p86)
	“Some of those poses, like opening the legs up or even stretching them, helped a little bit with my range of motion and it relaxed around my joints, especially the ones that hurt the most.” (Evans, 2011, p6)
1d	“I can see how yoga can correct your body position if you keep on doing that…I’m more aware of how I can do that with my muscles too; lifting up your leg to make it straighter instead of letting your legs do whatever they want.” (Evans, 2011, p6)
	“It made me feel better…overall it helped, just in general helped my energy level so that helped across the board with life.” (Evans, 2010, p910)
1e	“Halfway through this we reduced my dose from 15 to 7.5mgs so I’m on half the dose of methotrexate and I’m doing fine on it.” (Cartwright, 2020, p3)
2a	“I tried to do the exercise on Wednesday but I don’t think I did very well. I need to keep my mind only on what I am doing…I tried to do a ten minute meditation. I was concentrating on just one sentence. It was hard to stay focus. I tried just seeing it in my head like a tie on tape but was hard. I don’t know if I am doing this correctly.” (Middleton, 2017, p86)
2b	‘‘I’m older now and I needed to do something about balance and posture.’’ (Greysen, 2017, p490)
	“I try to do it when I exercise I can and I want to learn them and that way I won’t depend on the instructors, soon she will leave us alone and we will have to do it ourselves.” (Middleton, 2017, p86)
	‘‘I was pretty proud of myself for getting through it. when I do certain moves, I can feel pretty good about myself.’’ (Greysen, 2017, p491)
2c	“…God what a great thing to learn at a young age, because sometimes pain can be worsened because we’re thinking about it too much so if you are in a position to learn how to quiet that, it will help anything. If you can learn to do that when you are young, how much damage would you have in the long run?” (Evans, 2010, p910)
	‘‘A group of friends here at work had talked about doing Bikram yoga, and there was a deal, so I tried it.’’ (Greysen, 2017, p490)
2d	“I am very grateful for doing yoga. I am more tranquil and I can sleep more because I practice breathing and letting go of everything in my mind.” (Middleton, 2017, p86)
	“The strength you have within yourself to make a difference.” (Cartwright, 2020, p10)
3a	“It tuned me into coping, it tuned into my mind in how I deal with situations…I had to help myself which she taught me to do.” (Cartwright, 2020, p24)
3b	“My pain is still there…but now the difference is that I could reduce the pain by relaxing and just learning to be stress-free and just to be more peaceful. I’ve learned that if I’m peaceful and more stress free my pain eases away a little. So I’m doing better now.” (Evans, 2010, p910)
3c	“I felt like I found this inner peace within me. I found a side of myself that I didn’t know I had before…You let go of everything when you’re there doing yoga…I forgot about the pain sometimes.” (Evans, 2011, p7)
3d	“I think I need to stick with practicing yoga on a regular basis to improve symptoms long-term.” (Evans, 2010, p911)
4a	“I don’t do a lot of yoga because of time restraints.” (Cheung, 2022, p7)
	“My disabled daughter continues to [need]a great deal…my husband got prostate cancer…” (Cheung, 2022, p7)
4b	“It was really hard to bend my joints, I felt like it wasn’t for me or people with arthritis.’’ (Greysen, 2017, p490)
	“It gave me so much stress…[The therapist] was trying to find out, like you know like something like, something in my life which has caused this thing” (Cartwright, 2020, p10)
4c	“I wasn’t overwhelmed, so I wasn’t like, ’oh my gosh, this is too hard for me, I can’t go back,’ so, it was just my right pace.” (Greysen, 2017, p490)
	“Tailored the routine to suit me and how my health was at that time. If I couldn’t do something because of a certain movement then we’d take that bit out.” (Cartwright, 2020, p23)
4d	“But for the person to have an understanding and knowledge…and empathy and to know, gosh, this person’s got a problem with that joint; this is the alternative way of getting the same effect with this exercise.” (Ward, 2011, p218)
	“…the fear can be a problem. But as long as you trust the person that’s actually teaching you, that, you know, they’ve got your well-being, best interests.” (Ward, 2011, p218)
4e	“I don’t think I could do the yoga unless I had something beside me to help me. The chair yoga offers security for me.” (Park, 2011, p323)
	“Standing on the toes holding the wall, it is pretty good. I did the warm up looking at the book.” (Middleton, 2017, p86)
5a	“I feel like it’ll be more of a safe environment being that it’s a study for arthritis and everyone in the class may have some issues.” (Evans, 2011, p4)
	“…they’re not going to go [laugh], you know, what’s wrong with you? You know, they already know what’s wrong with ya…” (Ward, 2011, p218)
	“It was a bit like a counselling session…where we would find triggers as to what is going on in my life, in my mind that would have a negative impact on my health, so pinpointing those areas and working on that, I feel has had a massive difference.” (Cartwright, 2020, p23)
5b	“I just would be more motivated if it was with other people I knew, sort of understood how I felt, or, you know, like, yeah, I just want, wanna feel more comfortable with, even if they are strangers that they’ve got the same illness and stuff.” (Ward, 2011, p215)

1. Yoga, arthritis, and the body: The anticipated and experienced impacts of yoga on physical well-being influenced yoga practice in people with arthritis

This synthesised finding was derived from 30 findings that were interpreted into five categories, which addressed the perceived impacts of yoga on the “body”, i.e., physical well-being in arthritis. It highlighted how yoga affects physical symptoms of arthritis, thereby either hindering or facilitating yoga practice. Limited awareness of yoga’s benefits led to a fear of worsening symptoms, discouraging participation. However, noticeable improvements, including relief from arthritis symptoms, increased body awareness, and energy levels, motivated yoga practice. Many reported that these physical benefits encouraged them to maintain an active lifestyle by practising yoga, and some also mentioned reduced reliance on arthritis medication.

**Barriers**

- Yoga practice seemed “an unknown territory” that might aggravate arthritis symptoms

In this category, six findings (U) were aggregated, which showed that people with arthritis were apprehensive about practising yoga due to their limited awareness of yoga and thought that some yoga poses might be beyond their physical capabilities. Some described attempting yoga but giving up eventually as they felt it might add to their existing symptom burden in arthritis, e.g., worsening pain, flare-ups, and even effects on comorbidities (e.g., heart problems) or cause injuries.

1. Uncertainty about yoga’s benefits on arthritis symptoms discouraged yoga practice

Two findings (U) were aggregated to create this category, which described how some people with arthritis were unsure and, at times, uncertain about yoga’s benefits on their symptoms. One of them described that on some days, they would feel yoga alleviated their symptoms; on other days, they would not perceive any noticeable difference in their symptoms.

**Facilitators**

1. Experiencing relief from arthritis symptoms as a result of yoga practice

Thirteen findings (U) were combined to form this category. People with arthritis mentioned improvement in arthritis symptoms, including pain relief, improved flexibility and mobility, stronger muscles, and reduced swelling, ultimately making them feel at ease with their bodies.

1. Yoga seemed to elevate body awareness and physical vitality, translating into people’s daily lives

This category consisted of seven findings (U). It captured accounts of people with arthritis, suggesting that yoga may have elevated their energy levels and helped cultivate physical awareness, i.e., alertness on body posture, alignment, and flexibility. One of them appreciated that practising yoga made them internally aware of how stress can sway them towards the structural misalignment of the body (e.g., bad posture) and expanded their body consciousness. They expressed that these physical health benefits translated to their daily lives, as they generally felt stronger and motivated to maintain a healthy and physically active lifestyle despite their symptoms.

1. Yoga practice was perceived to reduce reliance on arthritis medication for pain relief

Two findings (U) were combined to create this category. A few individuals with arthritis shared that regularly practising yoga helped them manage their pain and allowed them to reduce their prescribed medication dosage slightly.

2. Yoga, arthritis, and the mind: Levels of motivation and perceived impact on mental well-being influenced yoga practice in people with arthritis

This finding was synthesised from four categories comprising 29 findings. It described the interconnection between mental well-being and intrinsic and extrinsic motivations, which work together to influence yoga practice in people with arthritis. Low levels of intrinsic motivation, such as low self-efficacy, self-assurance, and concentration, discouraged yoga practice in arthritis. High levels of intrinsic motivation, including personal interest, prioritising health, and a sense of accomplishment, encouraged participation in yoga. External motivations, including physicians’ recommendations, discounted sessions, improved comorbidities, and support from family and friends, also positively influenced yoga practice in people with arthritis. Staying mentally motivated to practise yoga and consequently practising it led to perceived improvements in mental well-being, which, in turn, further reinforced the motivation to continue practising yoga.

**Barriers**

- Wavering intrinsic motivations (concentration, self-efficacy, and self-assurance) negatively influenced yoga practice

Three findings (U) were merged to form this category, reflecting how the lack of intrinsic motivations among people with arthritis may hinder yoga practice. Those new to yoga practice described struggling with keeping their minds focused at the moment and were unsure about their yoga practice due to low levels of self-efficacy and self-assurance.

**Facilitators**

2b. High levels of intrinsic motivations (personal interest in yoga, prioritising health, self-efficacy, self-assurance, and self-confidence) facilitated yoga practice

Eleven findings (U) were combined to create this category, illustrating how being intrinsically motivated might encourage yoga practice in people with arthritis. In this category, some people described that they were motivated to practice yoga due to their rising concerns towards health as they grew older, and some were enthusiastic to practice yoga out of personal interest. The ease with which yoga could be practised (minimal equipment and supervision) was perceived to increase self-efficacy and self-assurance in people with arthritis to practice yoga. They added that the high levels of intrinsic motivations provided a sense of accomplishment and bolstered their self-confidence in coping with their condition through continued yoga practice.

2c. Extrinsic motivations (doctor’s advice, reduced price of yoga sessions, improvement in other health conditions, exposure to yoga, and peer/family support) positively influenced yoga practice in arthritis

This category included seven findings (1 C, 6U) describing the external influences that may motivate people with arthritis to practice yoga. These included yoga recommended by physicians to deal with pain, the provision of yoga at discounted prices, and perceived improvements in comorbidities (e.g., diabetes) were reported to encourage yoga practice in people with arthritis. Many expressed that awareness of, and exposure to, yoga and its benefits earlier in their lives would have equipped them mentally to deal with their pain in the long run. Support from friends and/or family was also cited as a motivation to practise yoga for arthritis.

2d. Perception of improvements in mental well-being could encourage yoga practice

This category included eight findings (U), which reflected on several perceived improvements in mental health due to yoga practice. They mentioned being able to “let go” of their worries, pain, and discomfort in arthritis and feeling calm and relaxed. Further, perceptions of improvements in their anxiety and depression symptoms were also generally reported. Many expressed feeling “happy” and experienced evident positive changes in their mood, which gave them the mental strength to deal with their condition.

3. Yoga, arthritis, and the mind-body impact: The experience of mind-body benefits of yoga fostered a positive outlook on coping with arthritis and encouraged ongoing engagement with yoga practice in people with arthritis

This synthesised finding aggregated 23 findings into four categories. It explored the perceptions of people with arthritis regarding how practising yoga deepened their understanding of the link between their body and mind and imparted a sense of overall well-being. These perceived intertwined mind-body benefits of yoga provided a sense of empowerment and enabled people to take charge of their condition by positively shifting their perspective towards coping with the physical and mental aspects of their condition.

**Facilitators**

- Practising yoga gave people with arthritis a sense of empowerment, i.e., the ability to take control of their condition

Five findings (U) were merged to create this category, which showed how people with arthritis perceived that yoga practice gave them a sense of agency to deal with their physical and mental health in arthritis. They mentioned that yoga strengthened their mind-body connection and the mind-body benefits were influenced by each other, i.e., improved physical health (reduced pain) led to improved mental health (stress relief and feeling calm) and vice versa.

3b. Yoga was appreciated as a beneficial coping strategy for arthritis

Twelve findings (U) were merged to form this category, which described the perceptions of people with arthritis on how yoga practice offers skill-based coping strategies to deal with the physical and mental aspects of the condition.

3c. Practising yoga changed the way people with arthritis viewed their condition, allowing them to rediscover themselves and regain a sense of normalcy in their lives

This category included three findings (U) illustrating how people with arthritis perceive yoga as a way to reconnect with themselves. They mentioned feeling a sense of calm and being able to distract their minds from the physical discomfort of arthritis. They added that the mind-body benefits enabled reconnection with their “self” (bodies and minds), making them more physically and mentally aware of their arthritis and changing their negative outlook towards dealing with the condition.

3d. Consistent yoga practice was perceived as essential for achieving long-term holistic benefits for arthritis

Three findings (U) constituted this category, which showed how people with arthritis felt that to experience long-term holistic benefits of yoga, it was important to practise yoga regularly and incorporate it into daily routines, including practice at home.

4. Yoga, arthritis, and session accessibility and structure: Engagement with yoga practice was influenced by individually determined factors affecting access to sessions, as well as by the structural characteristics of the sessions

This synthesised finding was generated from five categories developed from 22 findings. It described factors determining an individual’s access to yoga sessions (e.g., logistical considerations) and the structural characteristics of a yoga session (e.g., yoga poses delivered and their difficulty level, suitable modifications offered in the sessions, pace of the sessions, qualities and professional training of the yoga providers, and availability of props and resources) that influenced yoga practice in people with arthritis.

**Barriers**

- Juggling with logistical challenges to attending yoga sessions discouraged yoga practice

Five findings (U) were combined to create this category. Time constraints (e.g., difficulty prioritising yoga over other activities) and financial limitations (e.g., inability to afford yoga sessions) were cited as common barriers to yoga practice among people with arthritis. Other responsibilities, such as caregiver or family commitments, were also perceived to hinder yoga practice in people with arthritis.

4b. Unsatisfactory yoga experiences, including the difficulty level of the practices and discomfort, hindered yoga practice

This category consisted of two findings (U), which showed how the delivery of yoga sessions affected yoga practice. There was some description of yoga delivered in the sessions that was not modified adequately according to people’s specific needs in arthritis, which made it physically challenging for them to practice yoga. The intrusive nature of the yoga therapist in a yoga therapy session, leading to stress and discomfort, was also highlighted as a barrier to yoga practice.

**Facilitators**

4c. Yoga’s adaptability and well-paced sessions to suit people’s needs and preferences in arthritis were perceived to be helpful

Six findings (U) were aggregated to create this category. Many people expressed their satisfaction about yoga being adapted and paced according to their needs, preferences, and capabilities in the sessions and its added advantage as a non-invasive and non-pharmacological option in arthritis treatment. They described how practising yoga tailored to their needs enabled them to accept their physical limitations and be more compassionate towards themselves, instead of feeling pressurised to achieve a certain yogic pose that might not suit their bodies.

4d. Availability of a yoga provider with positive qualities, knowledge, and professional training facilitated yoga practice

Four findings (U) were grouped to create this category, highlighting the positive qualities of a yoga provider that drew people with arthritis towards yoga practice. People with arthritis emphasised the importance of yoga providers being empathetic and receptive towards individual needs and having adequate professional training to meet those needs safely.

4e. The provision of props and resources was perceived to offer confidence and security for yoga practice

Five findings (U) were combined to create this category, highlighting how the availability of props and resources served as facilitators to yoga practice in people with arthritis. Using props, such as a chair, to perform yoga poses provided support and safety, instilling confidence and a sense of security in their practice. In addition, guidance from resources such as DVDs and yoga instruction manuals made it easy for them to continue yoga practice, even outside the yoga sessions, at their convenience.

5. Yoga, arthritis, and the session environment: A supportive social environment in yoga sessions, characterised by a welcoming space and meaningful connections, encouraged yoga practice in people with arthritis

This synthesised finding was created from eight findings merged into two categories. It encompassed the positive impact of the social environment of yoga sessions, including safe, empathetic, non-judgmental, and supportive spaces that enabled people to form social connections, ultimately enhancing yoga practice in people with arthritis.

**Facilitators**

- Safe and supportive space (in group sessions) and a “therapeutic” space (in one-to-one sessions) were considered important for yoga practice

Three findings (U) were combined to form this category, which highlighted the perceptions of people with arthritis regarding the positive influence of the environment of a yoga session. The availability of a safe, non-judgmental, supportive, and inclusive space in the yoga sessions was commonly cited as a major motivation to practice yoga for arthritis. One of them went for yoga therapy sessions and appreciated the “therapeutic” element in a one-to-one setting where the yoga therapist would pay close attention to individual needs and provide tailored modifications.

5b. Social connectedness in a group setting was perceived as a strong motivation for yoga practice

Five findings (U) comprised this category. People with arthritis expressed that practising yoga in groups helped them connect with others having arthritis, strengthened their social bonds and fostered a sense of community, which reduced feelings of isolation.

## Discussion

This systematic review synthesised qualitative evidence on factors influencing yoga practice, with the categories within each synthesised finding offering insight into the barriers and facilitators to yoga practice in people living with arthritis. Six of the eight included studies met at least seven of the ten quality criteria, indicating moderate to high methodological quality. These factors related to (i) the anticipated and experienced impacts of yoga on physical well-being influenced yoga practice in people with arthritis; (ii) the levels of motivation and perceived impact on mental well-being influenced yoga practice in people with arthritis; (iii) the experience of mind-body benefits of yoga fostered a positive outlook on coping with arthritis and encouraged ongoing engagement with yoga practice in people with arthritis; (iv) engagement with yoga practice was influenced by individually determined factors affecting access to sessions, as well as by the structural characteristics of the sessions; and (v) a supportive social environment in yoga sessions, characterised by a welcoming space and meaningful connections, encouraged yoga practice in people with arthritis.

Overall, this review suggested that yoga can be a positive and empowering experience, articulating a range of facilitators to regular yoga practice, and therefore highlighting its potential as a complementary approach to arthritis treatment. Nevertheless, several important barriers were also identified in this review. A key concern was the apprehension among people with arthritis about yoga practice, as they thought yoga might be beyond their physical capabilities and could worsen their condition. Media portrayals often depict yoga as a physically intensive practice with a risk of injury, potentially discouraging yoga practice [48]. This review highlighted the need for future efforts to reduce fears surrounding yoga practice in people with arthritis.

Our review found that intrinsic motivations, e.g., self-efficacy, self-assurance, and self-confidence, can act as both barriers and facilitators to yoga practice in people living with arthritis. Low levels of intrinsic motivation were perceived as obstacles to yoga practice, whilst higher levels were perceived to facilitate it. Yoga practice was also perceived to enhance mental well-being, which in turn boosted intrinsic motivation, creating a positive self-sustaining cycle. This interconnected relationship highlighted the link between mental well-being and an individual’s motivation to practice yoga for arthritis. This aligned with findings from a systematic review on physical activity (including a wide range of physical activity interventions, including yoga, suggesting that self-driven intrinsic motivations (e.g., self-compassion) could significantly enhance well-being and further encourage physical activity [49]. This review highlighted improvement in arthritis symptoms, particularly pain relief, as a key motivator for practising yoga in arthritis. A narrative review suggested that yoga may improve pain tolerance and alleviate pain perception [50]. Further, systematic reviews have shown that yoga may help reduce joint stiffness and pain and improve function in osteoarthritis [25–28, 51]. In our own previous systematic reviews and meta-analysis of yoga interventions for osteoarthritis and rheumatoid arthritis, we found quantitative evidence for improvements in outcomes including pain, function, and disease activity score [25, 26]. The present review builds on this by synthesising qualitative insights into how people with arthritis themselves experience and perceive yoga, thus complementing the clinical outcomes literature with patient-centred perspectives. Our review supported these findings, also noting that reduced feelings of anxiety and depression as a result of yoga practice were perceived to make it easier for people with arthritis to cope with the mental burden of the condition. This reinforced evidence that yoga positively impacts mental health in rheumatic diseases, including arthritis [52]. A novel insight from this review was the perceived interplay between mind and body in yoga practice, leading to a sense of mind-body (holistic) benefits. The findings suggest that yoga was not viewed as merely a physical activity but as a practice also contributing to emotional and mental well-being. These synergistic, interconnected, and holistic benefits were a key facilitator underpinning yoga practice in people with arthritis. Accordingly, people with arthritis reported a changed outlook on their condition, a restored sense of normalcy, and a feeling of empowerment to take control of their condition. This resonated with findings from a qualitative study on patients’ perceptions of practising yoga to manage chronic pain, in which participants felt that yoga reframed their perceptions of living with it [53]. Therefore, the review emphasised the importance of recognising and promoting yoga as a holistic practice, encompassing physical poses, breathing practices, and meditation and relaxation practices to obtain overall benefits in arthritis.

In addition to health-related benefits, individually determined factors influencing access to yoga sessions, and several structural characteristics of the sessions were also perceived to influence yoga practice in people with arthritis. These included common barriers such as lack of time, financial constraints, and other responsibilities, similar to challenges reported in the general adult population [54]. While physical activity is essential for alleviating arthritis symptoms, people with arthritis can face particular barriers to staying active, poor adherence to exercise, limited guidance on injury prevention, and fears about exacerbating their condition [15, 55, 56]. These concerns can make it difficult to find a suitable form of physical activity [57]. Yoga may offer a valuable alternative, as it emphasises stretching, strength, posture, balance, and allows for adjustable pace and intensity, ensuring both health benefits and safety [58].

This review found that the adaptability of yoga (e.g., using props) and the flexible pacing of sessions to accommodate arthritis-related needs and capabilities were key facilitators for yoga practice. This finding aligned with qualitative studies emphasising yoga’s ability to be tailored to meet the evolving physical and mental health needs in specific health conditions, including arthritis [59–61]. Beyond the practice itself, our review also underscored the important characteristics of the yoga provider. People with arthritis valued empathetic yoga providers who encouraged acceptance of physical limitations, a finding echoed by qualitative studies exploring UK-based yoga providers’ insights on arthritis treatment [60]. While our earlier qualitative study focused on yoga provider insights [60], the present review focuses on the voices of people with arthritis, offering a complementary view that can inform the design of more acceptable and sustainable yoga programmes. Additionally, yoga sessions delivered by knowledgeable and professionally trained yoga providers capable of adapting practices to meet arthritis-specific needs were considered essential for ensuring safety and encouraging participation [60].

The social environment of yoga sessions was another key facilitator of yoga practice, synthesised in this review. A safe, supportive, and inclusive space where people did not feel judged due to their physical limitations was perceived as crucial to encourage yoga practice. People with arthritis often desire social interaction but withdraw from social activities due to their symptoms, leading to isolation [62, 63]. Group-based yoga sessions were perceived to engender social support and reduce feelings of loneliness in people with arthritis, thus facilitating yoga practice. These review findings were consistent with prior research suggesting that the sense of community and connection fostered in group yoga settings reduces isolation and offers social support [60, 64].

Overall, our findings suggest that future research should consider clear patient education strategies that highlight the adaptability of yoga, emphasise its physical, mental, and social benefits, and include practical guidance for safe home practice. Tailored handouts, using arthritis-specific modifications, could help reassure patients and increase accessibility across sociodemographic groups. Yoga providers delivering yoga to people living with arthritis should receive training on safety considerations, including yoga practices that may be contraindicated for individuals with arthritis, strategies to modify poses for varying levels of severity and comorbidities, and communication tips to boost people’s confidence in sustaining long-term practice. In addition, structured group support mechanisms, such as small class sizes and opportunities for people to share progress, may help sustain yoga practice and reduce isolation.

To our knowledge, this is the first systematic review to synthesise the barriers and facilitators to yoga practice in people with arthritis. The review followed JBI and PRISMA guidelines to ensure the methodology was robust. At least two independent reviewers were involved at each step to enhance credibility and minimise researcher and selection biases, and a record of decisions regarding how the synthesised findings and categories were created was maintained to further strengthen reliability. In line with JBI guidance, we adopted a reflexive approach by openly considering how our professional and social backgrounds and theoretical perspectives might shape the interpretive process. This reflexive stance was maintained throughout study selection, data extraction, and synthesis, to enhance transparency and minimise the influence of assumptions on the findings. Although our search strategy did not apply date or language restrictions and included grey literature sources (e.g., theses, dissertations, conference abstracts), it is possible that relevant unpublished studies or qualitative research published in other languages could have been missed. Consequently, the risk of publication and language bias cannot be completely eliminated. Such bias may have influenced the range of studies identified and the balance of qualitative insights represented in this review. Also, access to the EThOS database was not possible due to an ongoing cyber-attack, limiting the ability to retrieve relevant UK doctoral theses for this review. Only eight studies met our inclusion criteria, reflecting the limited availability of qualitative evidence in the evolving area of yoga and arthritis research. The lack of detail in the included studies limited the ability to fully assess their methodological quality, which may reduce the trustworthiness of the findings. Most studies had small sample sizes, involved predominantly women, and focused on osteoarthritis or rheumatoid arthritis, but not on other types of arthritis. This limits the generalisability of findings, particularly to male participants, people with other types of arthritis, different age groups, and people with comorbidities. All included studies were conducted in high-income Western countries (USA, UK, and New Zealand). This may not capture cultural and socio-economic insights on yoga, which is particularly important given its origins in South Asia, where it is formally recognised and widely practised, and the growing interest in yoga within low- and middle-income countries. Gender differences are also noteworthy, as existing evidence suggests women are more likely than men to practise yoga, underscoring the need to explore barriers and facilitators to yoga in more diverse cultural settings, socio-economic contexts, and gender groups to ensure that interventions are inclusive and globally relevant. At the same time, the concentration of studies in comparable high-income contexts may be viewed as a strength, as it enhances the transferability of our findings across these settings. This reflects what has been described as a multi-context qualitative review design, in contrast to context-specific syntheses [65]. In addition, reporting standards varied across studies. Few addressed reflexivity (i.e., how researchers’ own positions and assumptions may have influenced the research process) or used strategies such as member checking to verify participants’ accounts. These gaps raise questions about the credibility and transparency of findings. Furthermore, the included studies were short-term interventions, which restricts understanding of long-term adherence to yoga and the balance of its benefits and harms over time. Future research should therefore recruit more diverse populations, ensure clear reporting of reflexivity and verification, and explore yoga practice across cultural, socio-economic, and gender groups. Future studies should also explore the long-term sustainability of yoga practice for arthritis treatment, given the chronic nature of the condition.

## Conclusion

This review synthesised both barriers and facilitators to yoga practice in people with arthritis. Within the included studies, facilitators were somewhat more frequently reported than barriers, in line with the possibility that yoga could be a valuable addition to arthritis treatment. Key barriers included fear of injury, lack of motivation, and limited accessibility to yoga sessions. Physical, mental, and mind-body benefits of yoga were prominent facilitators. Further, yoga’s adaptability, availability of professionally trained and empathetic yoga providers, and supportive group environments also encouraged yoga practice in people with arthritis. Addressing these barriers and enhancing the facilitators can support the successful implementation of future yoga interventions in arthritis treatment.

## Supplementary Information

Below is the link to the electronic supplementary material.

Supplementary Material 1

## Data Availability

The data underlying this article are available in the article and its online supplementary material.

## Declarations

### Disclaimer

Neither commercial agencies were involved nor artificial intelligence (AI) was used in the drafting, writing, editing, or revision of the manuscript. The authors confirm that all content was prepared, reviewed, and approved by the authors themselves. No part of this manuscript is copied or published elsewhere, in whole or in part.

## References

[1] World Health Organization Musculoskeletal health. [revised 2022; cited 2024 Oct 30]. https://www.who.int/news-room/fact-sheets/detail/musculoskeletal-conditions

[2] Senthelal S, Li J, Goyal A et al (2023) Arthritis. [revised ; cited 2024 Oct 30]. In: StatPearls [Internet]. Treasure Island (FL): StatPearls Publishing; 2022. https://www.ncbi.nlm.nih.gov/books/NBK518992/

[3] Neogi T (2013) The epidemiology and impact of pain in osteoarthritis. Osteoarthr Cartil 21(9):1145–1153 10.1016/j.joca.2013.03.018 PMC3753584 23973124

[4] Hunter DJ, Schofield D, Callander E (2014) The individual and socioeconomic impact of osteoarthritis. Nat Rev Rheumatol 10(7):437–441 24662640 10.1038/nrrheum.2014.44

[5] Sharma A, Kudesia P, Shi Q et al (2016) Anxiety and depression in patients with osteoarthritis: impact and management challenges. Open Access Rheumatology: Res Reviews 8:103–113 10.2147/OARRR.S93516 PMC5098683 27843376

[6] Kłak A, Raciborski F, Samel-Kowalik P (2016) Social implications of rheumatic diseases. Reumatologia 54(2):73–78 27407283 10.5114/reum.2016.60216 PMC4918047

[7] Bitton R (2009) The economic burden of osteoarthritis. Am J Manag Care 15(8):230–235 19817509

[8] Hunter DJ, March L, Chew M (2020) Osteoarthritis in 2020 and beyond: a lancet commission. Lancet 396(10264):1711–1712 33159851 10.1016/S0140-6736(20)32230-3

[9] Matcham F, Scott IC, Rayner L et al (2014) The impact of rheumatoid arthritis on quality-of-life assessed using the SF-36: a systematic review and meta-analysis. Semin Arthritis Rheum 44(2):123–130 24973898 10.1016/j.semarthrit.2014.05.001

[10] Almutairi K, Nossent J, Preen D et al (2021) The global prevalence of rheumatoid arthritis: a meta-analysis based on a systematic review. Rheumatol Int 41(5):863–877 33175207 10.1007/s00296-020-04731-0

[11] Buelt A, Narducci DM (2021) Osteoarthritis management: updated guidelines from the American college of rheumatology and arthritis foundation. Am Family Phys 103(2):120–121 33448759

[12] National Institute for Health and Care Excellence (NICE) Rheumatoid arthritis: scenario: confirmed rheumatoid arthritis [Internet]. [revised 2025 April; cited 2025 May]. https://cks.nice.org.uk/topics/rheumatoid-arthritis/management/confirmed-ra/

[13] Singh JA, Wells GA, Christensen R et al (2011) Adverse effects of biologics: a network meta-analysis and Cochrane overview. Cochrane Database Syst Reviews 2. 10.1002/14651858.CD008794.pub2 10.1002/14651858.CD008794.pub2 PMC7173749 21328309

[14] Joensuu JT, Huoponen S, Aaltonen KJ et al (2015) The cost-effectiveness of biologics for the treatment of rheumatoid arthritis: a systematic review. PLoS ONE 10:e0119683 25781999 10.1371/journal.pone.0119683 PMC4363598

[15] Marks R (2012) Knee osteoarthritis and exercise adherence: a review. Curr Aging Sci 5(1):72–83 21762086 10.2174/1874609811205010072

[16] Collado-Mateo D, Lavín-Pérez AM, Peñacoba C et al (2021) Key factors associated with adherence to physical exercise in patients with chronic diseases and older adults: an umbrella review. Int J Environ Res Public Health 18(4). 10.3390/ijerph18042023 10.3390/ijerph18042023 PMC7922504 33669679

[17] Gilanyi YL, Shah B, Cashin AG et al (2024) Barriers and enablers to exercise adherence in people with nonspecific chronic low back pain: a systematic review of qualitative evidence. Pain 165(10):2200–2214 38635470 10.1097/j.pain.0000000000003234 PMC11404330

[18] Lasater J (1997) The heart of Patanjali. Yoga J 137:134–144

[19] Desikachar K, Bragdon L, Bossart C (2005) The yoga of healing: exploring yoga’s holistic model for health and well-being. Int J Yoga Therapy 15:17–39

[20] Zuckerman A Significant yoga statistics: 2020/2021 benefits, facts and trends. 2020. [cited 2024 Oct 20]. https://comparecamp.com/yoga-statistics/#TOC2

[21] Smith L 41 yoga statistics: how many people practice yoga? [revised 2023; cited 2024 Oct 23]. https://www.thegoodbody.com/yoga-statistics/

[22] Zhang Y, Lauche R, Cramer H et al (2021) Increasing trend of yoga practice among U.S. Adults from 2002 to 2017. J Altern Complement Med 27(9):778–785 34076530 10.1089/acm.2020.0506

[23] Anderson JG, Taylor AG (2011) The metabolic syndrome and mind-body therapies: a systematic review. J Nutr Metabolism 10.1155/2011/276419 10.1155/2011/276419 PMC3136147 21773016

[24] Kocyigit BF, Sagtaganov Z, Yessirkepov M (2023) The effectiveness of yoga as a form of exercise in the management of rheumatic diseases. Rheumatol Int 43(5):795–801 36856817 10.1007/s00296-023-05291-9

[25] Biswas I, Nalbant G, Lewis S et al (2024) Key characteristics of effective yoga interventions for managing osteoarthritis: a systematic review and meta-analysis. Rheumatol Int 44(9):1647–1677 38935121 10.1007/s00296-024-05652-y PMC11343886

[26] Biswas I, Kaur J, Pearce F et al (2025) Key features of effective yoga interventions in addition to standard medical treatment for rheumatoid arthritis: a systematic review and meta-analysis. ACR Open Rheumatol 7(5). 10.1002/acr2.70054 10.1002/acr2.70054 PMC12064992 40346985

[27] Lauche R, Hunter DJ, Adams J et al (2019) Yoga for osteoarthritis: a systematic review and meta-analysis. Curr Rheumatol Rep 21(9). 10.1007/s11926-019-0846-5 10.1007/s11926-019-0846-5 31338685

[28] Ye X, Chen Z, Shen Z et al (2020) Yoga for treating rheumatoid arthritis: a systematic review and meta-analysis. Front Med (Lausanne) 7:586665 33330545 10.3389/fmed.2020.586665 PMC7732597

[29] England BR, Smith BJ, Baker NA et al (2023) 2022 American college of rheumatology guideline for exercise, rehabilitation, diet, and additional integrative interventions for rheumatoid arthritis. Arthritis Rheumatol 75(8):1299–1311 37227071 10.1002/art.42507 PMC10947582

[30] Rausch Osthoff AK, Niedermann K, Braun J et al (2018) 2018 EULAR recommendations for physical activity in people with inflammatory arthritis and osteoarthritis. Ann Rheum Dis 77(9):1251–1260 29997112 10.1136/annrheumdis-2018-213585

[31] Cheung C, Wyman JF, Peden-McAlpine C (2022) Long-term yoga and aerobic/strength exercise adherence in older women with knee osteoarthritis: a mixed methods approach. Int Journey Yoga Therapy 32(4). 10.17761/2022-D-20-00033 10.17761/2022-D-20-00033 35405738

[32] Middleton KR, Magaña López M, Haaz Moonaz S et al (2017) A qualitative approach exploring the acceptability of yoga for minorities living with arthritis: ‘Where are the people who look like me?‘. Complement Ther Med 31:82–89 28434476 10.1016/j.ctim.2017.02.006 PMC5583513

[33] Evans S, Moieni M, Subramanian S et al (2011) Now I see a brighter day: expectations and perceived benefits of an Iyengar yoga intervention for young patients with rheumatoid arthritis. J Yoga Phys Therapy Rehabilitation 1(101). 10.4172/2157-7595.1000101 10.4172/2157-7595.1000101 PMC3493157 23145356

[34] Lockwood C, Porrit K, Munn Z et al (2020) Chapter 2: systematic reviews of qualitative evidence. In: Aromataris E, Munn Z (eds) JBI manual for evidence synthesis. JBI, Adelaide

[35] Tong A, Flemming K, McInnes E et al (2012) Enhancing transparency in reporting the synthesis of qualitative research: ENTREQ. BMC Med Res Methodol 12:181 23185978 10.1186/1471-2288-12-181 PMC3552766

[36] Page MJ, McKenzie JE, Bossuyt PM et al (2021) The PRISMA 2020 statement: an updated guideline for systematic reviews. PLoS Med 18(3):e1003583 33780438 10.1371/journal.pmed.1003583 PMC8007028

[37] School of Public Health, UTHealth. Search filters for various databases (2021) [cited 2024 Oct 13]. https://libguides.sph.uth.tmc.edu/search_filters/ovid_medline_filters

[38] Endnote X Clarivate Analytics, PA, USA. Endnote (2017) [cited 2024 Oct 30]. http://endnote.com/

[39] Mourad O, Hossam H, Zbys F et al (2016) Rayyan - a web and mobile app for systematic reviews. Syst Reviews 5:210 10.1186/s13643-016-0384-4 PMC5139140 27919275

[40] Munn Z, Aromataris E, Tufanaru C et al (2018) The development of software to support multiple systematic review types: the JBI system for the unified Management, assessment and review of information (JBI SUMARI). Int J Evid Based Healthc 10.1097/XEB.0000000000000152 30239357

[41] Lockwood C, Munn Z, Porritt K (2015) Qualitative research synthesis: methodological guidance for systematic reviewers utilizing meta-aggregation. Int J Evid Based Healthc 13(3):179–187 26262565 10.1097/XEB.0000000000000062

[42] Evans S, Moieni M, Taub R et al (2010) Iyengar yoga for young adults with rheumatoid arthritis: results from a mixed-methods pilot study. J Pain Symptom Manag 39(5):904–913 10.1016/j.jpainsymman.2009.09.018 20471550

[43] Cheung C, Justice C, Peden-McAlpine C (2015) Yoga adherence in older women six months post-osteoarthritis intervention. Global Adv Health Med 4(3):16–23 10.7453/gahmj.2015.041 PMC4424934 25984414

[44] Cartwright T, Cahill M, Sadana V (2020) A mixed methods evaluation of an individualised yoga therapy intervention for rheumatoid arthritis: pilot study. Complement Ther Med 50:102339 32444036 10.1016/j.ctim.2020.102339

[45] Greysen HM, Greysen SR, Lee KA et al (2017) A qualitative study exploring community yoga practice in adults with rheumatoid arthritis. J Altern Complement Med 23(6):487–493 28075155 10.1089/acm.2016.0156 PMC5488310

[46] Park J, McCaffrey R, Dunn D et al (2011) Managing osteoarthritis: comparisons of chair yoga, reiki, and education (pilot study). Holist Nurs Pract 25(6):316–326 22015342 10.1097/HNP.0b013e318232c5f9

[47] Ward L, Treharne GJ, Stebbings S (2011) The suitability of yoga as a potential therapeutic intervention for rheumatoid arthritis: a focus group approach. Musculoskelet Care 9(4):211–221 10.1002/msc.217 21800407

[48] Freeman H, Vladagina N, Ramji E et al (2017) Yoga in print media: missing the heart of the practice. Int J Yoga 10(3):160–166 29422747 10.4103/ijoy.IJOY_1_17 PMC5793011

[49] Ming W, Pak-Kwong C, Ka L (2021) The relationship between physical activity and self-compassion: a systematic review and meta-analysis. Mindfulness 12:547–563

[50] Chopra D, Stern E, Bushell WC et al (2023) Yoga and pain: a mind-body complex system. Front Pain Res (Lausanne) 4:1075866 36910253 10.3389/fpain.2023.1075866 PMC9996306

[51] Lu J, Kang J, Huang H et al (2024) The impact of yoga on patients with knee osteoarthritis: a systematic review and meta-analysis of randomized controlled trials. PLoS ONE 19(5):e0303641 38753745 10.1371/journal.pone.0303641 PMC11098307

[52] de Orleans Casagrande P, Coimbra DR, de Souza LC et al (2023) Effects of yoga on depressive symptoms, anxiety, sleep quality, and mood in patients with rheumatic diseases: systematic review and meta-analysis. Phys Med Rehabilitation 15(7):899–915 10.1002/pmrj.12867 35726183

[53] Tul Y, Unruh A, Dick BD (2011) Yoga for chronic pain management: a qualitative exploration. Scand J Caring Sci 25:435–443 21058970 10.1111/j.1471-6712.2010.00842.x

[54] Cagas JY, Biddle SJH, Vergeer I (2020) When an activity is more than just exercise: a scoping review of facilitators and barriers for yoga participation. Int Rev Sport Exerc Psychol 16(1):93–154

[55] Nogueira RMDR, de Souza Moura J, Costa CPS et al (2023) Adherence to exercise training and physical function in older adults diagnosed with knee osteoarthritis. Can Geriatr J 26(4):511–516 38045884 10.5770/cgj.26.674 PMC10684300

[56] Kanavaki AM, Rushton A, Efstathiou N et al (2017) Barriers and facilitators of physical activity in knee and hip osteoarthritis: a systematic review of qualitative evidence. BMJ Open 7(12):e017042 29282257 10.1136/bmjopen-2017-017042 PMC5770915

[57] InformedHealth.org [Internet]. Cologne, Germany: Institute for Quality and Efficiency in Health Care (IQWiG) (2006) Osteoarthritis of the knee: learn more – what can you do to strengthen your knees? [revised 2024; cited 2025 January 19]. https://www.ncbi.nlm.nih.gov/books/NBK544978/

[58] Mishra L, Parida N (2018) A current trend in the scenario of management of major joint disorder arthritis with yoga. Int J Adv Res 6. 10.21474/IJAR01/7127

[59] Vogler S, Salyer RE, Giacobbi PR (2023) Yoga and mental well-being: a qualitative exploration of the lived experiences of yoga practitioners. Int J Yoga 16(3):192–201 38463650 10.4103/ijoy.ijoy_191_23 PMC10919406

[60] Biswas I, Adebusoye B, Lewis S et al (2025) Clinical knowledge, experiences, and perceptions of yoga providers in arthritis treatment: a UK-based qualitative study. Rheumatol Int 45(4). 10.1007/s00296-025-05843-1 10.1007/s00296-025-05843-1 PMC11971125 40183922

[61] Atkinson NL, Permuth-Levine R (2009) Benefits, barriers, and cues to action of yoga practice: a focus group approach. Am J Health Behav 33(1):3–14 18844516 10.5993/ajhb.33.1.1

[62] Bay LT, Ellingsen T, Giraldi A et al (2020) To be lonely in your own loneliness: the interplay between self-perceived loneliness and rheumatoid arthritis in everyday life: a qualitative study. Musculoskelet Care 18(4):450–458 10.1002/msc.1480 32491275

[63] Nordkamp A, Midtgaard J, de Thurah A et al (2025) Excluding myself from what I need the most: experiences of loneliness in people with inflammatory arthritis: a qualitative study. Int J Rheum Dis 28(1):e70041 39800916 10.1111/1756-185X.70041 PMC11725708

[64] Ross A, Bevans M, Friedmann E et al (2014) I am A nice person when I do yoga!!! A qualitative analysis of how yoga affects relationships. J Holist Nurs 32(2):67–77 24166108 10.1177/0898010113508466 PMC4196270

[65] Hannes K, Harden A (2011) Multi-context versus context-specific qualitative evidence syntheses: combining the best of both. Res Synthesis Methods 2:271–278 10.1002/jrsm.55 26061890

