# Supplementary material for: Barriers and facilitators to yoga practice among people living with arthritis: a qualitative systematic review
Source: Rheumatol Int. 2025 Dec 17;46(1):18. doi: 10.1007/s00296-025-06037-5 (PMC12708792; doi:10.1007/s00296-025-06037-5)
Supplement: Supplementary file 1 — Supplementary Material 1 [file 296_2025_6037_MOESM1_ESM.docx]

**Appendix 1: Search strategies.**

**1. Medline (Ovid) Date of search: 07 Nov 2024;746 records**

1 exp Yoga/

2 exp Meditation/

3 exp Mind-Body Therapies/

4 "mind-body therap*".mp.

5 meditat*.mp.

6 (yoga* or yogi*).mp.

7 (asana* or pranayam* or dhyan* or ashtanga or bikram or hatha* or nidra or iyengar or kripalu or kundalini or vinyasa or raja or radja or bhakti or jnana or kriya* or karma or yama or niyama or pratyahara or dharana or samadhi or bandha or mudra* or chanda or sivananda).mp.

8 1 or 2 or 3 or 4 or 5 or 6 or 7

9 exp Arthritis/

10 (osteoarthr* or osteo-arthr*).mp.

11 (arthrit* or coxarthr* or chondrocalcinos#s or pseudogout or gout* or periarthriti* or oligoarthrit* or "r?eumatic fever*" or sacroiliit* or spondylarthrit*).mp.

12 "arthrosis deformans".mp.

13 (degenerative adj2 arthrit*).mp.

14 "degenerative joint disease*".mp.

15 (non-inflammatory adj3 arthrit*).mp.

16 ((inflammatory or idiopathic) adj2 (arthrit* or polyarthrit*)).mp.

17 ((r?eumat* or r?eumart* or revmatoid) adj3 (arthrit* or artrit* or diseas* or condition* or nodule* or factor*)).mp.

18 9 or 10 or 11 or 12 or 13 or 14 or 15 or 16 or 17

19 8 and 18

**2. Embase (Ovid) Date of search: 07 Nov 2024; 330 records**

1 exp alternative medicine/

2 exp yoga/

3 exp meditation/

4 “Mind body therap*”.mp.

5 (Yoga* or yogi*).mp.

6 Meditat*.mp.

7 (Asana* or Pranayam* or Dhyan* or Ashtanga or Bikram or Hatha* or Nidra or Iyengar or Kripalu or Kundalini or Vinyasa or Raja or Radja or Bhakti or Jnana or Kriya* or Karma or Yama or Niyama or Pratyahara or Dharana or Samadhi or Bandha or Mudra* or Chanda or Sivananda).mp.

8 1 or 2 or 3 or 4 or 5 or 6 or 7

9 exp Arthritis/

10 (osteoarthr* or osteo-arthr*).mp.

11 (arthrit* or coxarthr* or chondrocalcinos#s or pseudogout or gout* or periarthriti* or oligoarthrit* or "r?eumatic fever*" or sacroiliit* or spondylarthrit*).mp.

12 "arthrosis deformans".mp.

13 (degenerative adj2 arthrit*).mp.

14 "degenerative joint disease*".mp.

15 (non-inflammatory adj3 arthrit*).mp.

16 ((inflammatory or idiopathic) adj2 (arthrit* or polyarthrit*)).mp.

17 ((r?eumat* or r?eumart* or revmatoid) adj3 (arthrit* or artrit* or diseas* or condition* or nodule* or factor*)).mp.

18 9 or 10 or 11 or 12 or 13 or 14 or 15 or 16 or 17

19 (("semi-structured" or semistructured or unstructured or informal or "in-depth" or indepth or "face-to-face" or f2f or structured or guide*) adj3 (interview* or discussion* or questionnaire*)).ti,ab.

20 (focus group* or qualitative or ethnograph* or fieldwork or "field work" or "key informant").ti,ab.

21 exp interview/

22 **exp verbal communication/**

23 qualitative research/

24 19 or 20 or 21 or 22 or 23

25 8 and 18 and 24

**3. CINAHL Plus (EBSCOhost) Date of search: 07 Nov 2024; 52 records**

S1 (MH “Yoga+”)

S2 (MH “Mind Body Techniques+”)

S3 (MH “Meditation”)

S4 yoga* or yogi*

S5 “mind body therap*”

S6 meditat*

S7 asana* or pranayam* or dhyan* or ashtanga or bikram or hatha* or nidra or iyengar or kripalu or kundalini or vinyasa or raja or radja or bhakti or jnana or kriya* or karma or yama or niyama or pratyahara or dharana or samadhi or bandha or mudra* or chanda or sivananda

S8 S1 or S2 or S3 or S4 or S5 or S6 or S7

S9 (MH “Arthritis+”)

S10 osteoarthr* or osteo-arthr*

S11 arthrit* or coxarthr* or chondrocalcinos?s or pseudogout or gout* or periarthriti* or oligoarthrit* or "r#eumatic fever*" or sacroiliit* or spondylarthrit*

S12 “Arthrosis deformans”

S13 degenerative N2 arthritis

S14 degenerative joint disease*

S15 non-inflammatory N3 arthrit*

S16 (inflammatory or idiopathic) N2 (arthrit* or polyarthrit*)

S17 (r#eumat* or r#eumart* or revmatoid) N3 (arthrit* or artrit* or diseas* or condition* or nodule* or factor*)

S18 S9 or S10 or S11 or S12 or S13 or S14 or S15 or S16 or S17

S19 ("semi-structured" or semistructured or unstructured or informal or "in-depth" or indepth or "face-to-face" or f2f or structured or guide*) N3 (interview* or discussion* or questionnaire*)

S20 focus group* or qualitative or ethnograph* or fieldwork or "field work" or "key informant"

S21 S19 or S20

S22 S8 and S18 and S21

**4. PsycInfo (Ovid) Date of search: 07 Nov 2024; 87 records**

1 exp Mind Body Therapy/

2 “Mind-body therap*”.mp.

3 exp Yoga/

4 (Yoga* or yogi*).mp.

5 exp Meditation/

6 Meditat*.mp.

7 (Asana* or Pranayam* or Dhyan* or Ashtanga or Bikram or Hatha* or Nidra or Iyengar or Kripalu or Kundalini or Vinyasa or Raja or Radja or Bhakti or Jnana or Kriya* or Karma or Yama or Niyama or Pratyahara or Dharana or Samadhi or Bandha or Mudra* or Chanda or Sivananda).mp.

8 1 or 2 or 3 or 4 or 5 or 6 or 7

9 exp Arthritis/

10 (osteoarthr* or osteo-arthr*).mp.

11 (arthrit* or coxarthr* or chondrocalcinos#s or pseudogout or gout* or periarthriti* or oligoarthrit* or "r?eumatic fever*" or sacroiliit* or spondylarthrit*).mp.

12 "arthrosis deformans".mp.

13 (degenerative adj2 arthrit*).mp.

14 "degenerative joint disease*".mp.

15 (non-inflammatory adj3 arthrit*).mp.

16 ((inflammatory or idiopathic) adj2 (arthrit* or polyarthrit*)).mp.

17 ((r?eumat* or r?eumart* or revmatoid) adj3 (arthrit* or artrit* or diseas* or condition* or nodule* or factor*)).mp.

18 9 or 10 or 11 or 12 or 13 or 14 or 15 or 16 or 17

19 8 and 18

**5.** **AMED (Ovid) Date of search: 07 Nov 2024; 68 records**

1 Mind body medicine/

2 exp Yoga/

3 exp Meditation/

4 ("mind body" adj (medicine or therap*)).mp.

5 (Yoga* or yogi*).mp.

6 Meditat*.mp.

7 (Asana* or Pranayam* or Dhyan* or Ashtanga or Bikram or Hatha* or Nidra or Iyengar or Kripalu or Kundalini or Vinyasa or Raja or Radja or Bhakti or Jnana or Kriya* or Karma or Yama or Niyama or Pratyahara or Dharana or Samadhi or Bandha or Mudra* or Chanda or Sivananda).mp.

8 1 or 2 or 3 or 4 or 5 or 6 or 7

9 exp Arthritis/

10 (osteoarthr* or osteo-arthr*).mp.

11 (arthrit* or coxarthr* or chondrocalcinos#s or pseudogout or gout* or periarthriti* or oligoarthrit* or "r?eumatic fever*" or sacroiliit* or spondylarthrit*).mp.

12 "arthrosis deformans".mp.

13 (degenerative adj2 arthrit*).mp.

14 "degenerative joint disease*".mp.

15 (non-inflammatory adj3 arthrit*).mp.

16 ((inflammatory or idiopathic) adj2 (arthrit* or polyarthrit*)).mp.

17 ((r?eumat* or r?eumart* or revmatoid) adj3 (arthrit* or artrit* or diseas* or condition* or nodule* or factor*)).mp.

18 9 or 10 or 11 or 12 or 13 or 14 or 15 or 16 or 17

19 8 and 18

**6. Web of Science Date of search: 07 Nov 2024; 41 records**

**#1 ALL= (yoga* or “mind body therap*” or meditation or yogi* or asana* or pranayam* or dhyan* or meditat* or ashtanga or bikram or hatha* or nidra or iyengar or kripalu or kundalini or vinyasa or raja or radja or bhakti or jnana or kriya* or karma or yama or niyama or pratyahara or dharana or samadhi or bandha or mudra* or chanda or sivananda)**

**#2 ALL=((“Osteoarthritis” or osteoarthr* or osteo-arthr* or “coxarthrosis” or degenerative arthritis or degenerative joint disease* or non-inflammatory arthritis))**

**#3 ALL=(((r?eumat* or r?eumart* or revmatoid) N3 (arthrit* or artrit* or diseas* or condition* or nodule* or factor*)))**

**#4 ALL=((focus group* or interview* or narration or qualitative or ethnograph* or fieldwork or "field work" or "key informant"))**

**#5 #3 OR #2**

**#6 #5 AND #4 AND #1**

**7. ProQuest Dissertations and Theses (via Web of Science) Date of search: 07 Nov 2024; 6 records**

**#1 TS=((yoga* or mind body therap* or yogi* or asana* or pranayam* or dhyan* or meditat* or ashtanga or bikram or hatha* or nidra or iyengar or kripalu or kundalini or vinyasa or raja or radja or bhakti or jnana or kriya* or karma or yama or niyama or pratyahara or dharana or samadhi or bandha or mudra* or chanda or sivananda))**

**#2 TS=((osteoarthritis or osteoarthr* or osteo-arthr* or coxarthrosis or degenerative adj2 arthritis or degenerative joint disease* or non-inflammatory arthritis))**

**#3 TS=(("rheumatoid arthritis" OR "rheumatoid factor" or inflammatory arthritis OR idiopathic arthritis OR inflammatory polyarthritis))**

**#4 TS=((focus group* or qualitative or ethnograph* or fieldwork or "field work" or "key informant"))**

**#5 #2 OR #3**

**#6 #1 AND #4 AND #5**

**Appendix 2: Excluded studies with reasons for exclusion.**

**Ineligible phenomena of interest (n=3)**

Shariff F, Carter J, Dow C, et al. Mind and body management strategies for chronic pain and rheumatoid arthritis. Qualitative Health Research. 2009;19(8):1037-49.

Hipwell AE, Turner A, Barlow JH. Experiences of living with and self-managing arthritis: a qualitative exploration from Punjabi Sikh women’s perspectives. British Journal of Rheumatology. 2008;48(2):159.

Hawtin H, Sullivan C. Experiences of mindfulness training in living with rheumatic disease: an interpretative phenomenological analysis. British Journal of Occupational Therapy. 2011;74(3):137-42.

**Conference abstract (n=2)**

Sadana Vidhi, Cartwright T, Cahill M. A mixed methods evaluation of Viniyoga therapy intervention for rheumatoid arthritis. European Journal of Integrative Medicine. 2021;48:102081.

Greysen H. “It was just my right pace”: a qualitative study exploring yoga practice in adults with rheumatoid arthritis. In: [ACR/ARHP Combined Abstract Session: Rehabilitation](https://acrabstracts.org/sessions/acrarhp-combined-abstract-session-rehabilitation-2015) [Internet]. [2015 ACR/ARHP Annual Meeting](https://acrabstracts.org/meetings/2015-acrarhp-annual-meeting/): 2015 Nov 08; Arthritis and Rheumatology; 2015 Sep 09 [cited 2024 Nov 08]. Available from: https://acrabstracts.org/abstract/it-was-just-my-right-pace-a-qualitative-study-exploring-yoga-practice-in-adults-with-rheumatoid-arthritis/.

**Protocol (n=1)**

Middleton KR, Ward MM, Haaz S, et al. A pilot study of yoga as self-care for arthritis in minority communities. Health and Quality of Life Outcomes. 2013;11:10.1186/1477-7525-11-55.

**Appendix 3: Synthesised finding 1: Categories, findings, and illustrations.**

| Finding and credibility assigned | Illustration, study author, and page number | Category | **Yoga, arthritis, and the body:** The anticipated and experienced impacts of yoga on physical well-being influenced yoga practice in people with arthritis. |
| --- | --- | --- | --- |
| Hesitance regarding suitability due to unfamiliarity with yoga and being concerned about inability to perform certain poses. (U) | *“I saw people at gym doing it (yoga) and you’re like ‘yeah right’ I’m never going to be able to do that I’m not even gonna try….at first, I didn’t even consider it. You hear yoga and arthritis and you just don’t think the two mix.”* (Evans2011) (p4) | Yoga practice seemed “an unknown territory” that might aggravate arthritis symptoms. |  |
| Exercise preference. (U) | *"I stopped doing most of the floor yoga…getting up and down from the floor is more challenging."* (Cheung2022) (p7) |  |  |
| Physical concerns. (U) | *“I’m not gonna try yoga because I’m scared that it’s gonna make me have a flare-up and I’m gonna be in pain…”* (Ward2011) (p217) |  |  |
| Health status. (U) | *"I can do yoga, but I just have to be conscious about when I feel that flutter [from my heart valve problem]."* (Cheung2022) (p7) |  |  |
| Pain/discomfort. (U) | *"Today I practiced in the morning, I didn’t feel as motivated to do it. Because I had some pain. But afterwards I felt somewhat better.”* (Middleton2017) (p86) |  |  |
| Symptom burden – persistent flares. (U) | *‘‘I had to stop doing yoga because of my RA. I actually tried yoga again recently, but it caused a flare, so, I’ve got to stop doing that."* (Greysen2017) (p490) |  |  |
| Perception of no benefits. (U) | *“I don’t know that incorporating the yoga has greatly helped because I [already] do so much.”* (Cheung2022) (p8) | Uncertainty about yoga’s benefits on arthritis symptoms discouraged yoga practice. |  |
| Ambivalence about pain improvement. (U) | *"And in terms of the pain, I think some days I thought ‘‘Oh wow, it’s really working’’ and some days I don’t know. It’s hard to really tell."* (Evans2010) (p910) |  |  |
| Reduction of pain and improvement of movement. (U) | *"It makes my body feel better and I have less pain."* (Park2011) (p322) | Experiencing relief from arthritis symptoms as a result of yoga practice. |  |
| Physical benefit. (U) | *“ I am very happy because I have learned yoga, it is something new for me, it is helping me feel better, sleep better, to stretch and that helps me in my arthritis and I forget that I have it/suffer from it.”* (Middleton) (p86) |  |  |
| Good effects produced from yoga. (U) | *“Helps relieve the pain.”* (Cheung2022) (p6) |  |  |
| Physical benefit. (U) | *“I’ve been doing the yoga, and it really seems to be keeping the muscle tone…keeping it strong and in shape.”* (Cheung2022) (p6) |  |  |
| Less swelling. (U) | *“The swelling in my knee has gone down probably 80%, which is really good, and I attribute it to the yoga.”* (Cheung2022) (p33) |  |  |
| Less pain and developed stronger leg muscles and muscle tone. (U) | *“Mine [KOA] improved a lot…I really don’t complain now a lot about knee pain. I’ve developed more muscles in my legs, too, so that kind of helps me.”* (Cheung2022) (p33) |  |  |
| Yoga relieved OA symptoms. (U) | *“I think this stuff [yoga] really works. My pain is less, it helps me keep going. I am going to keep doing it.”* (Cheung 2015) (p21) |  |  |
| A sense of pain relief. (U) | *“I felt at ease with all my joints and my body.”* (Evans2010) (p910) |  |  |
| Physical health benefits – pain reduction. (U) | *“Pains started reducing and before it used to be eight, nine, level nine and then it started coming down three, four to two now or sometimes, some, sometimes it’s just gone completely like now, I’ve got nothing, no pain whatsoever.”* (Cartwright2020) (p21) |  |  |
| Physical benefit. (U) | *“Class was good today. I was on the mat for the first time. I feel I got a better workout on the mat. After we worked on our feet mine actually felt better. I seem to have more energy when I left class.”* (Middleton2017) (p86) |  |  |
| An improvement in morning symptoms. (U) | *“When I started, in the mornings, I would wake up with a little bit of pain in my elbow or knee or something and I really don’t have that at all anymore.”* (Evans2010) (p910) |  |  |
| Particular poses that had an action on corresponding body areas. (U) | *“Some of those poses, like opening the legs up or even stretching them, helped a little bit with my range of motion and it relaxed around my joints, especially the ones that hurt the most.”* (Evans2011) (p6) |  |  |
| Physical health benefits - improved sleep. (U) | *“I’m sleeping without pain because I’m not in pain, so I can lie down and just sleep and get up when the time comes to get up without being just exhausted for the whole day.”* (Cartwright2020) (p3) |  |  |
| Regular yoga practice helped participants become more conscious about their bodies and participate in other physical activities. (U) | *"I think about my posture more whether I am standing or sitting, even when I am doing dishes.”* (Cheung2015) (p21) | Yoga seemed to elevate body awareness and physical vitality translating into peoples’ daily lives. |  |
| Participants reported practising yoga along with other forms of exercise to stay healthy and active. (U) | *“ I have always been active. I bike, hike, and do Pilates. I now add yoga.”* (Cheung2015) (p21) |  |  |
| A sense of being more aware of body posture, alignment, and flexibility. (U) | *“I can see how yoga can correct your body position if you keep on doing that...I’m more aware of how I can do that with my muscles too; lifting up your leg to make it straighter instead of letting your legs do whatever they want.”* (Evans2011) (p6) |  |  |
| Increase in physical vitality generalised to many facets of living. (U) | *“(Yoga) just in general helped my energy level so that helped across the board with life.”* (Evans2011) (p5) |  |  |
| Awareness of long-term tension in the body and the development of a misaligned body posture to accommodate painful joints. (U) | *“I always feel like my body is caved inward. I’m always trying to protect my joints, and I’ve sort of learned and feel that after every class, I feel more open.”* (Evans2011) (p6) |  |  |
| Improved general functioning. (U) | *“It made me feel better .overall it helped, just in general helped my energy level so that helped across the board with life.”* (Evans2010) (p910) |  |  |
| An expanded awareness that extended from an internal knowledge of stress response to a structural awareness of how their bodies, including affected joints, may be misaligned. (U) | *“Never been that in tune with my body that I could feel the physical reaction of my stress.”* (Evans2011) (p6) |  |  |
| Reduction in medication dosage. (U) | *“Halfway through this we reduced my dose from 15 to 7.5mgs so I’m on half the dose of methotrexate and I’m doing fine on it.”* (Cartwright2020) (p3) | Yoga practice was perceived to reduce reliance on arthritis medication for pain relief. |  |
| Yoga helped reduce medication and still manage the pain. (U) | *‘‘get off one of the crutches’’* (Evans2010) (p910) |  |  |

**Appendix 4: Synthesised finding 2: Categories, findings, and illustrations.**

| Finding and credibility assigned | Illustration, study author, and page number | Category | **Yoga, arthritis, and the mind**: Levels of motivation and perceived impact on mental well-being influenced yoga practice in people with arthritis. |
| --- | --- | --- | --- |
| Self-efficacy: novice. (U) | *“I tried to do the exercise on Wednesday but I don’t think I did very well. I need to keep my mind only on what I am doing…I tried to do a ten minute meditation. I was concentrating on just one sentence. It was hard to stay focus. I tried just seeing it in my head like a tie on tape but was hard. I don’t know if I am doing this correctly.”* (Middleton2017) (p86) | Wavering intrinsic motivations (concentration, self-efficacy, and self-assurance) negatively influenced yoga practice. |  |
| Barriers - lack of concentration. (U) | *"I am also confused because when I relax in the moment that I am doing the exercise my mind starts to think about what I will be doing later.”* (Middleton2017) (p86) |  |  |
| Self-assurance. (U) | *"I question my own accuracy."* (Cheung2022) (p7) |  |  |
| Motivated to practice yoga because of one’s own enthusiasm or interest. (U) | *"I like [yoga in the water] so much that it motivates me to get out early in the morning, and I feel great."* (Cheung2022) (p6) | High levels of intrinsic motivations (personal interest in yoga, prioritising health, self-efficacy, self-assurance, and self-confidence) facilitated yoga practice. |  |
| Desire for physical fitness. (U) | *“I’m older now and I needed to do something about balance and posture.”* (Greysen2017) (p490) |  |  |
| Health was a greater priority than busy schedules. (U) | *“Prioritised and made time because it’s my health.”* (Cartwright2020) (p10) |  |  |
| Greater appreciation for the value of self-care. (U) | *“What’s number one is me and I need to look after myself.”* (Cartwright2020) (p10) |  |  |
| Self-efficacy: improvement. (U) | *“It was a very good experience because I didn’t need help. Although I am not sure it was perfect but just the experience, I hope to perfect it.”* (Middleton) (p86) |  |  |
| Self-efficacy: novice. (U) | *"I try to do it when I exercise I can and I want to learn them and that way I won’t depend on the instructors, soon she will leave us alone and we will have to do it ourselves."* (Middleton) (p86) |  |  |
| Self-efficacy: improvement. (U) | *“Practiced for 30 minutes. no Pain. I’m getting into a better flow with my home practice, but still need to use the manual.”* (Middleton2017) (p86) |  |  |
| Increased self-efficacy extended to physical activity. (U) | *”Every morning I try to stay more active now...even if I have some pain.”* (Evans2011) (p7) |  |  |
| Confidence in own ability to practice yoga. (U) | *“It’s easy, and there’s no equipment.”* (Cheung2022) (p7) |  |  |
| A sense of pride or accomplishment in their physical abilities and self-confidence. (U) | *“I was pretty proud of myself for getting through it. When I do certain moves, I can feel pretty good about myself.”* (Greysen2017) (p491) |  |  |
| Self-assurance. (U) | *"This is an activity that I actually can do. I use those home practice instruction sheet[s] we got for the study."* (Cheung2022) (p7) |  |  |
| There was a sense that if only they had known about yoga classes earlier in their diagnosis, they could have prevented much of the physical and psychological impairment they had experienced. (U) | *“If I had learned this even when I was in high school, God what a great thing to learn at a young age, because sometimes pain can be worsened because we’re thinking about it too much so if you are in a position to learn how to quiet that, it will help anything. If you can learn to do that when you are young, how much damage would you have in the long run?”* (Evans2010) (p910) | Extrinsic motivations (doctor’s advice, reduced price of yoga sessions, improvement in other health conditions, exposure to yoga, and peer/family support) positively influenced yoga practice in arthritis. |  |
| Reduced price of yoga classes. (U) | *“I won a free one-month pass to a yoga studio at the auction at my daughter’s school.”* (Greysen2017) (p489) |  |  |
| Suggestion of physician. (U) | *“When I got into the Pain Management Program, they suggested that I get into yoga.”* (Greysen2017) (p489) |  |  |
| Motivator-being influenced by others. (U) | *“A group of friends here at work had talked about doing Bikram yoga, and there was a deal, so I tried it.”* (Greysen2017) (p490) |  |  |
| Social/emotional support. (U) | *“Today I did standing poses, my son did them too, and he was also helping me by telling me how. Then I felt that gradually, the stretches are rather easy to do.”* (Middleton2017) (p86) |  |  |
| Measurable improvements to other conditions – diabetes, high blood pressure. (U) | *“I went to see the doctor a couple of weeks ago and she said your blood, we, we took a blood test before that so they were analysing my bloods, she said your diabetes has gone down, your blood pressure has gone down so I was really chuffed. I can only put it down to the therapy.”* (Cartwright 2020) (p3) |  |  |
| Perceived improvements to health strongly motivated the patients to continue with their yoga practice. (C) | *“It’s really inspired me to do the exercises regularly.”* (Cartwright2020) (p9) |  |  |
| Mental/emotional benefit. (U) | *"I am very grateful for doing yoga. I am more tranquil and I can sleep more because I practice breathing and letting go of everything in my mind."* (Middleton) (p86) | Perception of improvements in mental well-being could encourage yoga practice. |  |
| Mental/emotional benefit. (U) | *“When I do the exercises I feel good, I am thinking about what I am doing, I feel very centered in thought and spirit.”* (Middleton2017) (p86) |  |  |
| Great impact on emotional well-being and mood. (U) | *“It was very helpful for my mind more so than anything.”* (Evans2011) (p7) |  |  |
| Improvements in depressive and anxious symptoms. (U) | *“Before doing the yoga, I was having some troubles at home. Very depressing. So I was in bed a lot more…I just locked myself up and stayed in my room but now I’ve noticed that I’m outside of my room now, I’m out with my family, in the kitchen with my mom, I’m visiting my friends, I’m not staying home like I was before. I still get sad a little bit but it’s not like the depression that used to haunt me a lot more before…Now I see a brighter day. I’m looking forward to tomorrow, and the next day whereas before I wouldn’t even look.”* (Evans2011) (p8) |  |  |
| Benefits after the yoga program. (U) | *“My rheumatologist even noticed the change in my fatigue and mood…”* (Evans2010) (p911) |  |  |
| Significant increases in mood and mental well-being. (U) | *“I’m just happy and everybody can see that I’m happy.”* (Cartwright2020) (p9) |  |  |
| Mental reframing and taking control. (U) | *“The strength you have within yourself to make a difference.”* (Cartwright2020) (p10) |  |  |
| Focused breathing enabled them to reduce their anxiety in multiple situations and this transferability helped develop their resilience. (U) | *“When you do deep breathing your mind is somewhere else, you forget about your worries or whatever.”* (Cartwright2020) (p11) |  |  |

**Appendix 5: Synthesised finding 3: Categories, findings, and illustrations.**

| Finding and credibility assigned | Illustration, study author, and page number | Category | **Yoga, arthritis, and the mind-body impact:** The experience of mind-body benefits of yoga fostered a positive outlook on coping with arthritis and encouraged ongoing engagement with yoga practice in people with arthritis. |
| --- | --- | --- | --- |
| Yoga equipped participants with the tools to see themselves as efficacious and capable of managing their functioning. (U) | *“I just learned that always try new things, not to think you can’t do it and keep ongoing in life like that…don’t be scared…find a way that you can do it.”* (Evans2011) (p7) | Practising yoga gave people with arthritis, a sense of empowerment i.e., the ability to take control of their condition. |  |
| Rather than being a panacea for pain and symptom relief, yoga provided a tool to manage pain. (U) | *“My pain is still there…but now the difference is that I could reduce the pain by relaxing and just learning to be stress-free and just to be more peaceful. I’ve learned that if I’m peaceful and more stress free my pain eases away a little. So I’m doing better now.”* (Evans2010) (p910) |  |  |
| Benefits – self-management. (U) | *“…there’s no way we can control it but it’s, it’s just managing how we can control it. So, finding those techniques that can help us get through it or, you know.”* (Ward2011) (p215) |  |  |
| Increased agency arising from confidence in taking charge of health. (U) | *“I like to walk everywhere now.”* (Cartwright2020) (p9) |  |  |
| Self-management at home. (U) | *“No, it’s easier to do it at home,’ cause then you can choose your own time, you’re not getting there by 1 o’clock or 10 o’clock or whatever, you can please yourself when you do it.”* (Ward2011) (p216) |  |  |
| A sense of body awareness, mental clarity, and positive coping. (U) | *“It makes you aware of all the parts of your body in a sense…When I just exercised before, I didn’t think about how it all connects, and so there’s that feeling that it’s all connected in some way”* (Cheung2022) (p33) | Yoga was appreciated as a beneficial coping strategy for arthritis. |  |
| An improved sense of well-being. (U) | *"There is no question that these sessions have improved my sense of well-being, my psychological, my sociological, all of them."* (Park 2011) (p323) |  |  |
| Mind-body benefits. (U) | *“It just made me feel better, more flexible, calmer inside, stronger…It helps my mood, outlook and energy.”* (Greysen2017) (p490) |  |  |
| Beneficial for balance, posture, strength, breathing, stress, and sleeping patterns. (U) | *“I might get a feeling of calmness, and balance. Obviously stretching. Ah breathing is great for me cause I need that, and learning to centre your thoughts.”* (Ward2011) (p217) |  |  |
| Inner discipline. (U) | *“I definitely feel improvement with less pain, less stiffness. I eat better, I sleep better, my focus is much better, more disciplined for the things that I need to stay healthy for my RA.’’* (Greysen2017) (p490) |  |  |
| Yoga benefits. (U) | *"Yoga helps relaxation and improves balance…It’s a stress reliever…it helps you cope and clear your mind."* (Cheung2022) (p6) |  |  |
| Building coping skills. (U) | *“It tuned me into coping, it tuned into my mind in how I deal with situations…I had to help myself which she taught me to do.”* (Cartwright2020) (p24) |  |  |
| A coping tool for mental aspects of RA. (U) | *‘‘It helps mentally with acceptance that you have a disease, allowing it to be a part of your life. ..somebody with debilitating disease, we are kind of messed up mentally…it does something to your head and makes you feel weak and small...yoga changes the way you think about your RA.”* (Greysen2017) (p491) |  |  |
| Yoga helped cope with the negative emotions associated with RA. (U) | *“I think the breathing and the meditation are a big part…it’s just, when you have RA you can get a lot of negative thinking and this really helps with that…yoga is really helpful, like a tool to turn to.”* (Greysen2017) (p490) |  |  |
| Yoga provided a skill-based coping strategy for the patients, which contributed to their sense of confidence and self-efficacy. (U) | *“I learned that even if the joint hurts, even if my hips hurt, I can still do different poses, I can still relax them even if they’re hurting, I can do different things with my hips.”* (Evans2011) (p7) |  |  |
| Yoga provided an enhanced ability to relax and the time to be peaceful, away from stressors, and daily hassles. (U) | *“I really enjoyed it. I found it to be really relaxing and prior to this particular experience, I never knew how to relax. I’ve never had that moment of pure quiet, like a quiet mind because I live in my head. It was nice to find something that actually got me that quiet in my own head.”* (Evans2011) (p5) |  |  |
| Yoga was a tool for relaxation. (U) | *“Not just my knees, I feel I am more positive about things when I do yoga. The meditation and breathing help me relax.”* (Cheung 2015) (p21) |  |  |
| Yoga gave a different outlook on life and pain. (U) | *“I felt like I found this inner peace within me. I found a side of myself that I didn’t know I had before…You let go of everything when you’re there doing yoga…I forgot about the pain sometimes.”* (Evans2011) (p7) | Practising yoga changed the way people with arthritis viewed their condition, allowing them to rediscover themselves and regain a sense of normalcy in their lives. |  |
| A sense of enlightenment. (U) | *“felt like I was not a yoga person and now I can see anybody can be a yoga person.”* (Evans2011) (p6) |  |  |
| Rediscovery: ‘I’ve got my life back’ (U) | *“I feel my normal self. And I’ve been going out with friends and family now. We’re going to, this one’s having a party, birthday party, 60^th^ party, I’m gone. Before, no I can’t make it, I can’t, I’m too tired and but now it’s like I’ve got my life back in a sense.”* (Cartwright2020) (p3) |  |  |
| Yoga routine/habit. (U) | *“Every morning, class 5 days a week…"* (Cheung2022) (p6) | Consistent yoga practice was perceived essential for achieving long-term holistic benefits for arthritis. |  |
| Increased yoga adherence. (U) | *“Yoga is one of the things I’ve increased at home on my own.”* (Cheung2022) (p32) |  |  |
| Participants realized that to experience ongoing benefits, they would need to continue. (U) | *“I think I need to stick with practicing yoga on a regular basis to improve symptoms long-term.”* (Evans2010) (p911) |  |  |

**Appendix 6: Synthesised finding 4: Categories, findings, and illustrations.**

| Finding and credibility assigned | Illustration, study author, and page number | Category | **Yoga, arthritis, and session accessibility and structure:** Engagement with yoga practice was influenced by individually determined factors affecting access to sessions, as well as by the structural characteristics of the sessions. |
| --- | --- | --- | --- |
| Time. (U) | *"I don’t do a lot of yoga because of time restraints."* (Cheung2022) (p7) | Juggling with logistical challenges to attending yoga sessions discouraged yoga practice. |  |
| Time. (U) | *“So I would prefer to even do it on the way home, or late afternoon, or, you know, almost at some part of my working day…”* (Ward2011) (p217) |  |  |
| Cost. (U) | *"…a lot of people with rheumatoid arthritis are on invalid benefits and things like that, and that often, even though if they’re not paying out other things for their health, it’s often a barrier because there’s, you know, living costs and that; you don’t get that much, you know."* (Ward2011) (p215) |  |  |
| Cost. (U) | *“I would try it more if they offered it as a part of your health plan membership.’’* (Greysen2017) (p490) |  |  |
| Caregiver responsibilities. (U) | *"My disabled daughter continues to [need]a great deal…my husband got prostate cancer…"* (Cheung2022) (p7) |  |  |
| Barrier – class difficulty. (U) | *“It was really hard to bend my joints, I felt like it wasn’t for me or people with arthritis.”* (Greysen2017) (p490) | Unsatisfactory yoga experiences, including the difficulty level of the practices and discomfort, hindered yoga practice. |  |
| Talking to the yoga therapist in therapy sessions was not useful or appropriate. (U) | *“It gave me so much stress…[The therapist] was trying to find out, like you know like something like, something in my life which has caused this thing”* (Cartwright2020) (p10) |  |  |
| Programme structure. (U) | *"Yoga is noncompetitive and accepting. It offers modifications."* (Cheung2022) (p6) | Yoga's adaptability and well-paced sessions to suit people’s needs and preferences in arthritis were perceived to be helpful. |  |
| Benefits – ‘Yoga meets you where you are’’. (U) | *“I wasn’t overwhelmed, so I wasn’t like, ’oh my gosh, this is too hard for me, I can’t go back,’ so, it was just my right pace.”* (Greysen2017) (p490) |  |  |
| Benefits – non-invasive, non-pharmacological. (U) | *“…it looks pretty non-invasive, so it looks like it shouldn’t be too many problems.”* (Ward2011) (p215) |  |  |
| Benefits – Adaptable – postures, breathing, relaxation. (U) | *“…say I had a flare-up and I went along, I’d probably say, can I sit in a chair and just do breathing exercises.”* (Ward2011) (p215) |  |  |
| Personally tailored practice: ‘a different kind of yoga’. (U) | *“Tailored the routine to suit me and how my health was at that time. If I couldn’t do something because of a certain movement then we’d take that bit out.”* (Cartwright2020) (p23) |  |  |
| Yoga taught with the support of tools and tailored modifications seemed to bypass physical limitations and participants could be more forgiving. (U) | *“I learned to accept my body and not just stay at home in bed if I’m in pain.”* (Evans2011) (p7) |  |  |
| Participants made it clear that having a ‘textbook’ knowledge of RA was not enough in a teacher – they also wanted someone who could empathize with what it was like to live with a chronic illness. (U) | *"But for the person to have an understanding and knowledge…and empathy and to know, gosh, this person’s got a problem with that joint; this is the alternative way of getting the same effect with this exercise"* (Ward2011) (p218) | Availability of a yoga provider with positive qualities, knowledge, and professional training facilitated yoga practice. |  |
| Developing trust in the teacher would enable them to overcome the fear that many of them experienced as an outcome of their current RA management. (U) | *“…the fear can be, you know, a problem. But as long as you trust the person that’s actually teaching you, that, you know, they’ve got your well-being, best interests.”* (Ward2011) (p218) |  |  |
| Programme instructions. (U) | *“Having an instructor that understands and is concerned about your joints really helped.”* (Cheung2022) (p6) |  |  |
| Participants were appreciative of the careful selection of therapeutic sequences and the inclusion of modifications and teachers trained to work with students with health needs. (U) | *"It just seemed different because…somehow with the regular yoga, it seems like it’s all about ‘Look, how strong I am; look at these interesting positions I can make my body into.’ Whereas this seemed more like they actually cared about your health and how to make you feel better, and you know, let’s try and get you into a position that’s going to help you and relax you at the same time, which seemed a lot more pleasant."* (Evans2011) (p5) |  |  |
| Props and resources. (U) | *“I stretch my legs, my arms, whole body, I cannot balance but I grab a chair.”* (Middleton) (p86) | The provision of props and resources was perceived to offer confidence and security for yoga practice. |  |
| Sense of security of having a chair. (U) | *“I don’t think I could do the yoga unless I had something beside me to help me. The chair yoga offers security for me."* (Park 2011) (p323) |  |  |
| Resources as a potential facilitator. (U) | *"…something that would really work for me and probably would keep me going would be something like a DVD that I could do at home on a daily basis."* (Ward2011) (p216) |  |  |
| Props as a potential facilitator. (U) | *“They would need to supply, like, a bench to lie on and chairs to sit on and things like that."* (Ward2011) (p216) |  |  |
| Props and resources. (U) | *“Standing on the toes holding the wall, it is pretty good. I did the warm up looking at the book."* (Middleton2017) (p86) |  |  |

**Appendix 7: Synthesised finding 5: Categories, findings, and illustrations.**

| Finding and credibility assigned | Illustration, study author, and page number | Category | **Yoga, arthritis, and the session environment:** A supportive social environment in yoga sessions, characterised by a welcoming space and meaningful connections, encouraged yoga practice in people with arthritis. |
| --- | --- | --- | --- |
| Yoga provided a space where no one judged their ability to perform the yoga practice and where participants gained social support from people who understood their condition. (U) | *“…they’re not going to go [laugh], eww, you know, what’s wrong with you? You know, they already know what’s wrong with ya…”* (Ward2011) (p218) | Safe and supportive space (in group sessions) and a “therapeutic” space (in one-to-one sessions) were considered important for yoga practice. |  |
| A tailor-made yoga intervention for people with RA gave them the courage to attempt yoga. (U) | *“I feel like it’ll be more of a safe environment being that it’s a study for arthritis and everyone in the class may have some issues.”* (Evans2011) (p4) |  |  |
| The therapeutic function of the consultation: ‘like counselling’. (U) | *“It was a bit like a counselling session...where we would find triggers as to what is going on in my life, in my mind that would have a negative impact on my health, so pinpointing those areas and working on that, I feel has had a massive difference.”* (Cartwright2020) (p23) |  |  |
| Family/social support. (U) | *“Do it with people…if you have a companion to exercise you’re more motivated.”* (Cheung2022) (p6) | Social connectedness in a group setting was perceived as a strong motivation for yoga practice. |  |
| Group: Commonality, applicability, adaptability/social support. (U) | *“I just would be more motivated if it was with other people I knew, sort of understood how I felt, or, you know, like, yeah, I just want, wanna feel more comfortable with, even if they are strangers that they’ve got the same illness and stuff.”* (Ward2011) (p215 ) |  |  |
| Social. (U) | *“Yoga was amazing to me, how much strength and energy you build up and there’s a social aspect of it…we’ve developed pretty good friendships there.”* (Greysen 2017) (p491) |  |  |
| Motivator-being influenced by others. (U) | *“A group of friends here at work had talked about doing Bikram yoga, and there was a deal, so I tried it.”* (Greysen2017) (p490) |  |  |
| Social/emotional support. (U) | *“Today I did standing poses, my son did them too, and he was also helping me by telling me how. Then I felt that gradually, the stretches are rather easy to do."* (Middleton2017) (p86) (RA) |  |  |
